# Risk factors for erectile dysfunction in diabetes mellitus: a systematic review and meta-analysis

**DOI:** 10.3389/fendo.2024.1368079

**Published:** 2024-04-04

**Authors:** Diliyaer Dilixiati, Alapati Waili, Aizihaier Tuerxunmaimaiti, Liwen Tao, Abudureheman Zebibula, Mulati Rexiati

**Affiliations:** ^1^ Department of Urology, First Affiliated Hospital of Xinjiang Medical University, Urumqi, China; ^2^ Department of Pancreatic Surgery, First Affiliated Hospital of Xinjiang Medical University, Urumqi, China; ^3^ Department of Cardiac Surgery, First Affiliated Hospital of Xinjiang Medical University, Urumqi, China

**Keywords:** diabetes mellitus, erectile dysfunction, risk factors, meta-analysis, sexual dysfuction

## Abstract

**Background:**

Previous studies have established that diabetes mellitus (DM) markedly raises the risk of developing erectile dysfunction (ED). Despite extensive investigations, the risk factors associated with ED in diabetic men have yet to be unequivocally determined, owing to incongruent and inconclusive results reported in various studies.

**Objective:**

The objective of this systematic review and meta-analysis was to assess the risk factors for ED in men with DM.

**Methods:**

A comprehensive systematic review was conducted, encompassing studies published in the PubMed, Scopus and Embase databases up to August 24th, 2023. All studies examining the risk factors of ED in patients with DM were included in the analysis. To identify significant variations among the risk factors, odds ratios (ORs) and their corresponding 95% confidence intervals (CIs) were employed. The risk of bias was evaluated using the Newcastle-Ottawa Scale(NOS) for longitudinal studies and the Agency for Healthcare Research and Quality Scale(AHRQ) for cross-sectional studies.

**Results:**

A total of 58 studies, including a substantial participant pool of 66,925 individuals diagnosed with DM, both with or without ED, were included in the meta-analysis. Mean age (OR: 1.31, 95% CI=1.24-1.37), smoking status (OR: 1.32, 95% CI=1.18-1.47), HbA1C (OR: 1.44, 95% CI=1.28-1.62), duration of DM (OR: 1.39, 95% CI=1.29-1.50), diabetic neuropathy (OR: 3.47, 95% CI=2.16-5.56), diabetic retinopathy (OR: 3.01, 95% CI=2.02-4.48), diabetic foot (OR: 3.96, 95% CI=2.87-5.47), cardiovascular disease (OR: 1.92, 95% CI=1.71-2.16), hypertension (OR: 1.74, 95% CI=1.52-2.00), microvascular disease (OR: 2.14, 95% CI=1.61-2.85), vascular disease (OR: 2.75, 95% CI=2.35-3.21), nephropathy (OR: 2.67, 95% CI=2.06-3.46), depression (OR: 1.82, 95% CI=1.04-3.20), metabolic syndrome (OR: 2.22, 95% CI=1.98-2.49), and diuretic treatment (OR: 2.42, 95% CI=1.38-4.22) were associated with increased risk factors of ED in men with DM.

**Conclusion:**

Our study indicates that in men with DM, several risk factors for ED have been identified, including mean age, HbA1C, duration of DM, diabetic neuropathy, diabetic retinopathy, diabetic foot, cardiovascular disease, hypertension, microvascular disease, vascular disease, nephropathy, depression, metabolic syndrome, and diuretic treatment. By clarifying the connection between these risk factors and ED, clinicians and scientific experts can intervene and address these risk factors, ultimately reducing the occurrence of ED and improving patient management.

## Introduction

Diabetes mellitus stands as a prevalent and formidable non-communicable disease that profoundly impacts the health and well-being of individuals, their families, and broader societies. DM represents a substantial global burden, exerting a significant impact on morbidity and mortality rates, and stands as the ninth leading cause of death worldwide (1). Epidemiological investigations have revealed a remarkable upsurge in the prevalence and mortality associated with DM from 2007 to 2017 (2). Projections suggest that by 2030, an estimated 10.2% of the global population will be affected by this chronic condition (3).

ED refers to the repetitive or persistent inability to attain and/or sustain an adequate level of erectile function required for satisfactory sexual intercourse (4). In individuals with DM, this condition typically emerges from the intricate interplay of neurogenic, vasogenic, and psychological factors, which are closely interlinked with the chronic complications related to DM (5). The prevalence of DM has rapidly increased due to higher consumption of high-sugar diets and decreased physical activity as a result of social development. The prevalence of ED among diabetic patients exhibits significant variation, spanning from 35% to 90% (6). Furthermore, in the United States, the total direct cost of evaluating ED treatment is estimated to be $400 million, with approximately a quarter of this amount linked to DM and obesity (7).

A recent study examined the association between DM and ED, treatment options, and diabetes-related ED, incorporating 106 relevant studies in the review (8). This extensive inclusion of studies highlights the widespread interest and significance of the association between DM and ED as a current and highly pertinent topic. Men with DM often contend with several comorbidities that serve as independent risk factors for ED, including advancing age, obesity, smoking, cardiovascular disease(CVD), hypertension, metabolic syndrome, and dyslipidemia (9, 10). In addition, the presence of diabetic complications such as diabetic retinopathy and diabetic foot can further precipitate the development of ED (5). The effects of ED reach far beyond physical symptoms, encompassing significant psychosocial and clinical implications. These implications are linked to men’s social interactions, emotional and psychological well-being, as well as their relationships with their partners. Nevertheless, it is important to highlight that ED stands as one of the most treatable complications of DM, with a success rate exceeding 95% in treatment outcomes (11).

ED is a prevalent complication of DM and high-quality meta-analyses and ED guidelines (12) have recognized DM as a significant risk factor. However, there remains a notable gap in the literature regarding a comprehensive analysis and synthesis of the various risk factors associated with ED in men affected by DM. Thus, we conducted a comprehensive exploration of the risk factors for ED in the diabetic population, aiming to furnish clinicians and preventive physicians with valuable insights for averting the onset of ED.

## Materials and methods

This meta-analysis adheres to the 2020 guidelines outlined in the Preferred Reporting Items for Systematic Reviews and Meta-Analyses (PRISMA) (13). The study protocol has been registered with the international prospective register of systematic reviews (PROSPERO) under the registration number CRD42023495323.

### Search strategy

A comprehensive systematic review was conducted, encompassing studies published in the PubMed, Scopus and Embase databases up to August 24th, 2023. Relevant studies derived from the references of the studies included in the initial search, along with significant reviews and systematic reviews pertinent to this field, were also comprehensively assessed to ensure comprehensive coverage of the literature. By utilizing a combination of medical subject heading (MeSH) terms and text words, we devised a preliminary search strategy that incorporated the following terms: “Diabet”; “insulin”; “resistance glucose”; “Intolerant”; “diabetes mellitus”; “T1DM”; “T2DM”; “Erectile Dysfunction”; “Impotence”. The comprehensive search strategy employed for all databases can be found in **Supplementary Material 1**.

### Study selection criteria

Three researchers (AW, AT, and L-WT) independently assessed all articles for eligibility and cross-validated their findings. Any discrepancies were resolved through discussion or consultation with the senior authors (DD).The inclusion criteria for the selected articles were as follows: (1) diagnosis of DM was conducted by either a specialist clinician, a qualified health manager, or through analysis of database data adhering to internationally recognized diagnostic criteria. (2) studies investigating risk factors for ED in men with DM. (3) studies involving male participants aged 18 years or older, and publications in English, irrespective of study design(longitudinal or cross-sectional). And (4) any studies that provided OR, relative risks (RR), hazard ratios (HR) with 95% CIs, or sufficient data to facilitate the calculation of these values. The following exclusion criteria were applied: (1) no control group was established in the study; (2) reviews, letters, conference abstracts, case reports, case series, or editorials; (3) duplicates, animal studies, non-English articles, or articles for which full-text access could not be obtained were excluded. When multiple articles from a single study reported on the same endpoint, only the data representing the longest follow-up period were extracted. Furthermore, in studies that reported multivariate adjusted effects, we extracted results from models that controlled for the most significant potential confounders. In cases where studies did not report an effect result or where data could only be extracted from baseline, we computed the effect result using a fourfold table and defined the result as unadjusted.

### Data extraction and quality assessment

Data extraction from each article was performed by three independent observers (AW, AT, and L-WT). Any discrepancies were resolved either by a third observer (DD) or through consensus among the observers. The extracted data included the first author’s name, year of publication, country of origin, study design, sample size, number of participants, mean age, ascertainment of DM and ED, type of DM, pharmaceutical treatments, outcomes, and other relevant factors.

The quality and methodological robustness of the included longitudinal studies were assessed by three researchers (AW, AT, and L-WT) using the Newcastle-Ottawa Scale (NOS) (14). As for the cross-sectional studies, these three researchers utilized the guidelines provided by the Agency for Healthcare Research and Quality (AHRQ) to evaluate their methodological rigor (15).

### Statistical analyses

Data analysis was performed using STATA software version 12.0 (STATA Corporation, Texas, USA). The primary outcome of this study examines the risk factors for ED in diabetic patients, while the secondary outcome focuses on conducting subgroup analyses to stratify risk factors based on the type of DM, age, and other relevant factors. Given the substantial representation of cross-sectional studies, ORs were used as effect sizes, and findings from a combination of longitudinal and cross-sectional studies were integrated to enhance the generalizability of our study, drawing upon insights from previous research endeavors (16, 17). Additionally, we conducted separate subgroup analyses to rigorously examine the results of studies, thereby enhancing the robustness of our findings. Heterogeneity in the study was evaluated through Cochrane’s Q test and I^2^ statistics. The fixed-effect model was adopted when P ≥ 0.1 and I^2^ ≤ 50%, while the random-effect model was utilized for cases when P < 0.1 and I^2^ > 50%. Further subgroup analyses were conducted using a comprehensive dataset including more than 10 studies to investigate potential causes of heterogeneity. We conducted sensitivity analyses by excluding individual studies and assessing their impact on the overall pooled results. Furthermore, we performed funnel plot analysis and evaluated publication bias using the Egger tests. Statistical significance was defined as a p-value less than 0.05 for all two-sided statistical tests.

## Results

**Figure 1:**
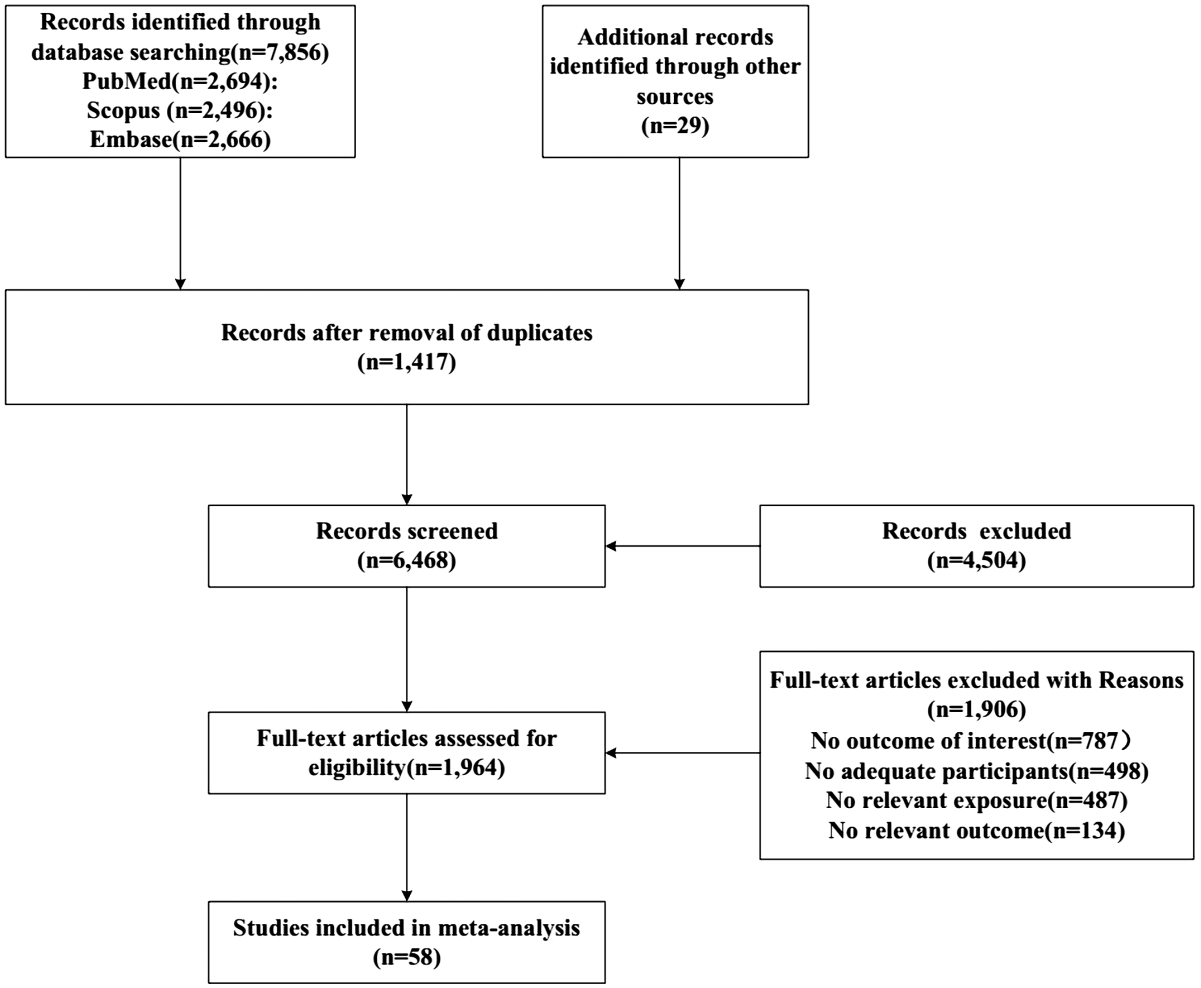
Flow chart of study selection.

The initial search involved a systematic review of a vast array of 7,885 studies, including 2,694 from PubMed, 2,496 from Scopus, and 2,666 from Embase. Furthermore, an additional 29 studies were identified through alternative sources. After the removal of 1,417 duplicated studies and the exclusion of an additional 4,504 based on the evaluation of their title and abstract content, a rigorous assessment was conducted on 1,964 studies for full-text evaluation. Finally, 58 articles met the criteria for inclusion in the meta-analysis and literature review. The process employed to identify eligible articles is depicted in **Figure 1**.

Of the 58 studies ultimately included, a comprehensive analysis revealed a total of 37 identified risk factors, encompassing the following categories: demographic and lifestyle characteristics (mean age, BMI, weight, waist-to-hip ratio, alcohol consumption, smoking status, lower income, physical activity, sitting time); laboratory analyses (systolic blood pressure, low HDL cholesterol, estimated glomerular filtration rate, testosterone levels, Hemoglobin, microalbuminuria); diabetes-related complications (HbA1c levels, inadequate glycemic control, duration of DM, diabetic neuropathy, diabetic retinopathy, Diabetic foot); and medical history and symptomatology (CVD, hypertension, microvascular disease, vascular disease, nephropathy, depression, premature ejaculation, atherogenic dyslipidemia, reduced libido, metabolic syndrome, hyperuricemia, nocturia, cardiorespiratory fitness diuretics, ACE inhibitors, injectable insulin).

### Study characteristics

During the conclusive analysis, 22 of the 37 identified risk factors were subjected to meta-analysis, indicating that they were each supported by a minimum of two included studies. As there was an insufficient number of eligible studies, a meta-analysis could not be conducted on the 15 risk factors. Therefore, **Table 1** presents the raw data extracted from the individual articles that were included in this study. All included articles, spanning from 1996 to 2023, encompassed a total cohort of 66,925 participants. Among the studies, 9 were conducted in Europe, 26 in Asia, 10 in North America, 1 in South America, 2 in Oceania, and 10 in Africa. The mean age of subjects ranged from 18.0 to 78.8 years. **Table 2** shows the characteristics of the included articles and the quality of each. **Table 1** presents the results of a meta-analysis or original outcome analysis that evaluates the influence of 37 identified risk factors on the occurrence of ED in men with DM.

**Table 1:** Categorical analysis on the correlation between risk factors for erectile dysfunction and diabetes mellitus.

Category of risk factors	No. of studies	OR(95%CI)	P	Heterogeneity	
				I^2^	P
Mean age	43	1.31(1.24,1.37)	<.001	94.8	<.001
BMI	16	0.96(0.88,1.05)	0.396	88.8	<.001
Weight	1	1.60(1.20,2.20)	–	–	-
Waist to Hip ratio	1	1.04(1.01,1.08)	–	–	-
Alcohol consumption	2	0.91(0.70,1.18)	0.486	64.3	0.061
Smoking status	16	1.32(1.18,1.47)	<.001	61.1	<.001
Lower income	1	2.16(1.32,3.52)	–	–	-
Physical activity	3	0.62(0.37,1.02)	0.059	84.6	0.002
Sitting time	1	4.57(1.46,18.03)	–	–	–
Systolic BP	1	1.02(1.01,1.04)	–	–	–
Low HDL cholesterol	2	8.86(3.64,21.57)	<.001	0	0.556
eGFR	1	0.99(0.97,1.00)	–	–	–
Testosterone	2	1.63(0.11,25.60)	0.727	95.1	<.001
Hemoglobin	1	0.83(0.69,0.99)	–	–	–
Microalbuminuria	2	3.77(1.98,7.18)	<.001	0	0.974
HbA1C	26	1.44(1.28,1.62)	<.001	87.0	<.001
Inadequate glycemic control	1	2.14(1.91,7.44)	–	–	–
Duration of DM	30	1.39(1.29,1.50)	<.001	95.2	<.001
Diabetic neuropathy	4	3.47(2.16,5.56)	<.001	72.1	0.013
Diabetic retinopathy	4	3.01(2.02,4.48)	<.001	75.6	0.006
Diabetic foot	2	3.96(2.87,5.47)	<.001	0	0.519
Cardiovascular disease	3	1.92(1.71,2.16)	<.001	25.3	0.262
Hypertension	28	1.74(1.52,2.00)	<.001	63.3	<.001
Microvascular disease	16	2.14(1.61,2.85)	<.001	77.8	<.001
Vascular disease	3	2.75(2.35,3.21)	<.001	0	0.627
Nephropathy	3	2.67(2.06,3.46)	<.001	30.0	0.240
Depression	4	1.82(1.04,3.20)	0.037	87.7	<.001
Premature ejaculation	1	4.41(2.08,9.39)	–	–	–
Atherogenic dyslipidemia	2	1.11(0.83,1.49)	0.477	46.0	0.173
Reduced libido	1	4.38(1.39,13.82)	–	–	–
Metabolic syndrome	4	2.22(1.98,2.49)	<.001	0	0.443
Hyperuricemia	1	2.89(1.02,8.16)	–	–	–
Nocturia	1	1.60(0.83,3.16)	–	–	–
Cardiorespiratory fitness	1	0.61(0.47,0.78)	–	–	–
Diuretic treatment	2	2.42(1.38,4.22)	0.002	68.6	0.075
ACE Inhibitors	1	0.80(0.21,3.07)	–	–	–
Insulin injectable	1	2.11(1.11,3.99)	–	–	–

OR, odds ratio; CI, Confidence interval; P, P value; I^2^, Information Gain Ratio.

**Table 2:** Characteristics of studies included in the meta-analysis.

Author	Country	Medication for DM	Sample size	Design	Mean age	Methods of ED ascertainment	Kind of DM	Factors	Variables adjusted or matched in the regression models	Study population	NOS/AHRQ scores
Abeway S 2020 (18)	Ethiopia	A	323	cross-sectional	>18	IIEF	Both	Age/Duration of DM/Insulin injectable	Age, Duration of DM diagnosis, types of medication, types of complication and body mass index	HB	10
Al-Hunayan, A. 2007 (19)	The State of Kuwait	NA	323	cross-sectional	65 ± 4.5	IIEF	DM2	Age/Smoking status/Hypertension/BMI	NA	HB	10
Almigbal, T. H. 2019 (20)	Kingdom of Saudi Arabia	A	293	cross-sectional	>18	IIEF	DM2	Age/Duration of DM	Age/Duration of DM	HB	8
Almigbal, T. H. 2018 (21)	Kingdom of Saudi Arabia	B	309	cross-sectional	60.2	IIEF	DM2	Age	Age	HB	7
Bortolotti A 2001 (22)	Italy	NA	9670	cross-sectional	65.7 ± 7.2	Enquiry	Both	Smoking status	Smoking status	HB	9
Burke, J. P. 2007 (23)	USA	NA	2115	Cohort study	69 ± 4.8	Enquiry	NA	Age	Age	HB	6
Chaudhary,R.K. 2013 (24)	China	NA	175	Cohort study	NA	Enquiry	DM2	Age/Hypertension	NA	HB	5
Chew,KK 2009 (25)	Australia	NA	4228	cross-sectional	NA	IIEF	NA	Hypertension	NA	HB	7
Chew,S.K 2013 (26)	Australia	NA	324	Cohort study	65.2 ± 8.6	IIEF	DM2	Age/HbA1c/BMI/Smoking status/Hypertension	Age/HbA1c/BMI/Smoking status/Hypertension	HB	8
Chuang, Y. C. 2012 (27)	China	NA	455	cross-sectional	58.9 ± 10.3	SHIM	DM2	Albuminuria/Hypertension/Systolic BP/eGFR/Hemoglobin/Albumin	Age, Duration of DM	HB	9
BaconCG 2002 (28)	USA	NA	2,057	Cohort study	65.8	IIEF	NA	Age/Duration of DM	NA	CB	8
D Fedele 1998 (29)	Europe	NA	9868	cross-sectional	NA	IIEF	Both	Age/Duration of DM	Age, Duration of DM	HB	10
Demir, T. 2008 (30)	USA	NA	62	Cohort study	54.2 ± 7.3	IIEF	DM2	Duration of DM/BMI/HbA1c/Hypertension	NA	HB	7
ElSaghier, E. O. A. 2015 (31)	Egypt	NA	70	cross-sectional	50.7 ± 4.04	IIEF	DM2	Age/HbA1c/BMI	NA	HB	8
Musa E 2021 (32)	Nigeria	B	358	cross-sectional	46.34 ± 5.66	IIEF	DM2	Age/Hypertension/BMI/Testosterone	NA	HB	10
Fedele D 2000 (33)	Italy	NA	9868	cross-sectional	69 ± 7.2	Enquiry	Both	Metabolic control/Arteriopathy/Cardiopathy/Nephropathy/Autonomic Neuropathy/Sensory-motor Neuropathy/Diabetic foot/Retinopathy/Duration of DM/BMI/Smoking status/Alcohol consumption	age	HB	11
FurukawaS 2017 (34)	Japan	C	430	Cohort study	60.7 ± 11.5	SHIM	DM2	Sitting time	Age, BMI, Duration of DM, diabetes, current Smoking status, current drinking, Hypertension, Dyslipidemia, coronary artery disease, glycated hemoglobin, walking habit,and diabetic Neuropathy	CB	6
FurukawaS 2016 (35)	Japan	A	332	Cohort study	57.1 ± 10.0	SHIM	DM2	Nocturia	Age, BMI,Hypertension, Dyslipidemia, stroke, ischemic heart disease, glycated, hemoglobin, current drinking, current Smoking status, use of insulin, use of oral antihyperglycemic agent and diabetic Neuropathy	CB	7
FurukawaS 2017 (36)	Japan	A	287	Cohort study	54.0 ± 8.9	SHIM	DM2	Diabetic Neuropathy	Age, BMI, Duration of DM, current, Smoking status, current drinking, Hypertension, Dyslipidemia, coronary, artery disease, stroke, glycated hemoglobin, diabetic Neuropathy, diabetic Retinapathy and diabetic Neuropathy	CB	7
FurukawaS 2017 (37)	Japan	A	469	Cohort study	60.9 ± 11.5	SHIM	DM2	Depressive symptoms	Age, BMI, waist, Duration of DM, current Smoking status, current drinking,Hypertension, Dyslipidemia, coronary artery disease, stroke, glycated hemoglobin and diabetic Neuropathy	CB	6
García-MalpartidaK. 2011 (38)	Spain	A	154	cross-sectional	55.9 ± 8.1	IIEF	DM2	Age/Duration of DM/Hypertension	Duration of DM condition	HB	8
GiuglianoF 2010 (39)	South Italy	NA	555	cross-sectional	57.9 ± 6.9	IIEF	DM2	Age/Duration of DM/Metabolic syndrome/BMI/WHR/Hypertension/Atherogenic Dyslipidemia/Physical activity/Depression	NA	HB	8
Gobena, M. B. 2023 (40)	Ethiopia	NA	210	cross-sectional	54.53 ± 13.73	IIEF	DM2	Age/Inadequate glycemic control	NA	HB	9
Habibi,A. 2011 (41)	Iran	NA	171	cross-sectional	52.78	IIEF	DM2	Depression/HbA1c/lower of HDL	NA	HB	7
Henis, O. 2011 (42)	Israel	NA	102	cross-sectional	64.0 ± 8.2	SHIM	DM2	DR severity	NA	HB	6
Hurisa, A.D. 2020 (43)	Ethiopia	NA	350	cross-sectional	47.9 ± 12.2	IIEF	Both	Age/Duration of DM	NA	HB	9
Jamieson, F. 2008 (44)	USA	NA	142	cross-sectional	40 ± 59	Enquiry	DM1	Age/Duration of DM/Microalbuminuria/HbA1c/Weight	NA	HB	7
Kalter-Leibovici, O. 2005 (45)	Israel	NA	1040	cross-sectional	57.0± 11.8	IIEF	DM1	Age/Duration of DM/HbA1C/MicroVascular disease/Cardiovascular disease/Diuretic treatment/Physical activity/Alcohol consumption	NA	HB	9
Kamenov, Z. A. 2007 (46)	Bulgaria	NA	150	cross-sectional	53± 12.5	IIEF	Both	Duration of DM/Retinapathy/Symptoms of DN	Age	HB	7
Katsimardou, A. 2023 (47)	The Hellenic Republic	A	45	cross-sectional	62.8 ± 10.54	IIEF	DM2	HDL	NA	HB	9
Klein R 1996 (48)	USA	NA	365	Cohort study	37.6 ± 11	Enquiry	DM1	History of current use/Age/Hb1Ac/BMI/AntiHypertension medication	Age	CB	7
Klein, R 2005 (49)	USA	C	264	Cohort study	34.4 ± 8.4	Enquiry	DM1	Age/Hypertension/Smoking status	NA	CB	5
Lo, W. H. 2014 (50)	China	NA	603	cross-sectional	60.5 ± 10.5	IIEF	DM2	Age/subjects who thought they had ED	NA	HB	8
Lu, C. C. 2009 (51)	China	NA	792	cross-sectional	65.6 ± 13.2	SHIM	DM2	Age/Duration of DM/Hypertension/Dyslipidemia/Smoking status/Hb1Ac	Age, Duration of DM	HB	8
Malavige, L. S. 2008 (52)	Sri Lanka	NA	253	cross-sectional	55.6 ± 10.4	IIEF	DM2	PE/Reduced libido/Lower income/Age/Duration of DM	NA	HB	9
Zeleke M 2021 (53)	Ethiopia	A	352	cross-sectional	49.14 ± 13.047	IIEF	DM2	Duration of DM/Age/Hypertension/Hb1Ac/BMI	NA	HB	8
Minami, H. 2018 (54)	Japan	A	460	Cohort study	60.8 ± 11.6	SHIM	DM2	Higher PA	NA	HB	7
Miyata Y 2004 (55)	Japan	A	226	cross-sectional	55.8 ± 10.1	IIEF	DM2	Age/Hb1Ac/Smoking status/BMI	NA	HB	8
Mutagaywa, R. K. 2014 (56)	The United Republic of Tanzania	NA	312	cross-sectional	51.33 ± 15.03	IIEF	DM2	Age/Neuropathy/Vascular disease	NA	HB	6
Naya Y 2003 (57)	Japan	NA	647	cross-sectional	43.6	IIEF	DM2	Age/Hypertension	NA	CB	8
Ndang Ngou Milama, S. 2022 (58)	Gabon	C	333	cross-sectional	56.6 ± 9.8	IIEF	DM2	Duration of DM/Age/Hypertension/Hyperuricemia/Hb1Ac/Smoking status/BMI	NA	HB	9
Nisahan, B. 2019 (59)	Sri Lanka	C	326	cross-sectional	49 ± 7.5	IIEF	DM2	Duration of DM/Age/Hypertension/Hb1Ac/Smoking status/BMI	NA	HB	8
Nutalapati, S. 2020 (60)	India	A	720	cross-sectional	58.4 ± 7.8	IIEF	DM2	Duration of DM/Age/Hypertension/Hb1Ac/Testosterone	NA	HB	7
P. K. Moulik 2002 (61)	UK	C	499	cross-sectional	57 ± 15	IIEF	DM2	Duration of DM/Age/Hypertension/Hb1Ac/Smoking status	NA	HB	9
Palmer, M. R. 2017 (62)	USA	C	600	Cohort study	43.7 ± 6.3	IIEF	DM1	Duration of DM/Age/Hypertension/Hb1Ac/Smoking status/BMI	NA	HB	8
Pitta RM 2023 (63)	Brazil	NA	2047	cross-sectional	63.85	IIEF	DM2	Age/Hypertension	NA	CB	10
Rosen, R. C. 2009 (64)	USA	NA	373	Cohort study	45-75	IIEF	DM2	Age/Hb1Ac/Hypertension/Metabolic syndrome/Cardiorespiratory fitness	NA	HB	8
Sasaki, H. 2005 (65)	Japan	C	1118	cross-sectional	59 ± 8.0	IIEF	DM2	Age/Hypertension/Hb1Ac	Age/Hypertension/Hb1Ac	CB	5
Seid, A 2017 (66)	Ethiopia	C	249	cross-sectional	43.39	IIEF	DM2	Duration of DM/Age/Hypertension/Smoking status	NA	HB	6
Shiri, R. 2006 (67)	Iran	A	312	cross-sectional	47.8 ± 8.3	IIEF	DM2	Duration of DM/Age/Hypertension/Hb1Ac/Smoking status/BMI	Duration of DM/Age/Hypertension/Hb1Ac/Smoking status/BMI	HB	7
Siu, S. C. 2001 (68)	China	A	486	cross-sectional	58.3 ± 10.4	IIEF	DM2	Duration of DM/Age/Hypertension/Hb1Ac/Smoking status	NA	HB	8
TridiantarK 2020 (69)	Indonesia	C	122	cross-sectional	52.3 ± 6.4	IIEF	DM2	Duration of DM/Age/Hypertension/Hb1Ac	NA	HB	8
Van Cauwenberghe, J. 2022 (70)	Belgium	A	237	Cohort study	61 ± 9	IIEF	DM2	Duration of DM/Age/Hypertension/Hb1Ac	NA	CB	7
Walle, B. 2018 (71)	Ethiopia	A	422	cross-sectional	45.7	IIEF	DM2	Duration of DM/Age/Hypertension	Duration of DM/Age/Hypertension	HB	8
Weinberg, A. E. 2013 (72)	USA	A	3306	cross-sectional	48.23 ± 4.55	IIEF	DM2	Duration of DM/Age/Hb1Ac	NA	CB	6
Wessells, H. 2011 (73)	USA	C	761	Cohort study	48 ± 5.9	IIEF	DM1	Duration of DM/Age/Hb1Ac	NA	HB	10
Yang, G. 2010 (74)	China	A	5477	cross-sectional	54.2 ± 11.5	IIEF	DM2	Duration of DM/Age/Hypertension/Hb1Ac/Smoking status/BMI	NA	HB	6
Zheng, H. 2006 (75)	China	A	327	cross-sectional	50.7 ± 11.5	IIEF	DM2	Duration of DM/Age/Hypertension/Hb1Ac/Smoking status/BMI	NA	HB	7

A, Oral hypoglycaemic agents and Insulin; B, Metformin-glimepiride combination C, Insulin; DM2, Type 2 diabetes’; DM1, Type 1 diabetes; IIEF, International Index for Erectile Function; HB, Hospital base; CB, Community base. NA, Not Available.

### Demographic and lifestyle characteristics

The meta-analysis encompassed 43, 16, 2, 16, and 3 studies investigating the mean age, BMI, alcohol consumption, smoking status, and physical activity factors, respectively. Among these, mean age (OR: 1.31, 95% CI=1.24-1.37) and smoking status (OR: 1.32, 95% CI=1.18-1.47) were identified as significant risk factors, while BMI, alcohol consumption, and physical activity did not show significance(P ≥.05). Significant heterogeneity is present in both the mean age factor and smoking status factor(I^2^ = 94.8% and 64.1%, respectively). Furthermore, we observed a significant publication bias in relation to the mean age factor (Egger’s test: P <.001). However, when employing the trim and filling method, the results remained stable after applying the necessary adjustments. Additionally, the smoking status factor displayed no significant bias (Egger’s test: P = .631).The results of all meta-analyses involving demographic and lifestyle characteristics factors are presented in **Supplementary Figures 1**-**12**.

### Laboratory analyses

low HDL cholesterol, testosterone, and microalbuminuria factors were analyzed in two separate articles for meta-analysis. The results revealed no significant heterogeneity between low HDL cholesterol and microalbuminuria factors(I^2^ = 0% and 0%, respectively), prompting the utilization of the fixed-effect model. This model yielded significant results, indicating that both low HDL cholesterol(OR: 8.86, 95% CI=3.64-21.57) and microalbuminuria(OR: 3.77, 95% CI=1.98-7.18) were substantial risk factors. However, no discernible association was found between testosterone and the occurrence of ED(P = .727). The results of all meta-analyses involving Laboratory analyses factors are presented in **Supplementary Figures 13**-**15**.

### Diabetes-related complications

Meta-analyses were conducted on the factors of HbA1C(OR: 1.44, 95% CI=1.28-1.62), Duration of DM(OR: 1.39, 95% CI=1.29-1.50), Diabetic neuropathy(OR: 3.47, 95% CI=2.16-5.56), Diabetic retinopathy(OR: 3.01, 95% CI=2.02-4.48), and Diabetic foot(OR: 3.96, 95% CI=2.87-5.47), with a total of 26, 30, 4, 4, and 2 studies included, respectively. The findings demonstrated that these factors were substantiated as risk factors associated with an increased occurrence of ED in diabetic men. Significant heterogeneity was detected among the factors of HbA1C, Duration of DM, Diabetic neuropathy, and Diabetic retinopathy(I^2^ = 87.0%, 95.2%,72.1% and 75.6%, respectively), while no significant heterogeneity was observed for the Diabetic foot factor(I^2^ = 0%). Evidence of publication bias was identified in the studies examining the HbA1C and Duration of DM factors, as indicated by the results of the Egger’s test (P = 0.024, <.001, respectively). However, when employing the trim and filling method, the results remained stable after applying the necessary adjustments. The results of all meta-analyses involving Diabetes-related complications factors are presented in **Supplementary Figures 16**-**26**.

### Medical history and symptomatology

The meta-analysis conducted on CVD(OR: 1.92, 95% CI=1.71-2.16), hypertension(OR: 1.74, 95% CI=1.52-2.00), microvascular disease(OR: 2.14, 95% CI=1.61-2.85), vascular disease(OR: 2.75, 95% CI=2.35-3.21), nephropathy(OR: 2.67, 95% CI=2.06-3.46), depression(OR: 1.82, 95% CI=1.04-3.20), atherogenic dyslipidemia(OR: 2.22, 95% CI=1.98-2.49), metabolic syndrome(OR: 2.22, 95% CI=1.98-2.49), and diuretic treatment(OR: 2.42, 95% CI=1.38-4.22) revealed that these factors pose a significant risk for ED in diabetic men. Analyses of factors such as hypertension, microvascular disease, depression, and diuretic treatment exhibited considerable heterogeneity(I^2^ = 63.3%, 77.8%,87.7% and 68.6%, respectively). Conversely, analyses of factors such as CVD, vascular disease, nephropathy, atherogenic dyslipidemia, and metabolic syndrome demonstrated no significant heterogeneity. As a result, fixed-effect models were employed in these cases(I^2^ = 25.3%, 0%,30.0%, 46.0%, and 0%, respectively). No evidence of publication bias was detected in the results pertaining to the hypertension and microvascular disease factors(P = .527, = .296, respectively). The results of all meta-analyses involving medical history and symptomatology factors are presented in **Supplementary Figures 26**-**38**.

### Subgroup analysis and sensitivity analyses

Ration could possibly serve as a contributing factor to the observed heterogeneity in BMI, HbA1C, and microvascular disease factors. Furthermore, the utilization of medication for DM may be a potential source of heterogeneity in the relationship between smoking status factors, hypertension factors, and the development of ED. Lastly, the study design employed could be a plausible source of heterogeneity in the associations between BMI, smoking status, and microvascular disease factors. Significantly, we observed a notably higher incidence of ED within the African subgroup of the diabetic population, particularly in relation to mean age(OR: 2.38, 95% CI=1.52-5.26), duration of DM(OR: 3.16, 95% CI=1.41-7.08), and hypertension(OR: 2.23, 95% CI=1.50-3.31) factors. In addition, sensitivity analysis showed that our findings were reliable.

## Discussion

Our study, a Comprehensive Systematic Review and Meta-Analysis, has shed light on the multitude of risk factors associated with ED in men with DM. Notably, we have identified several key risk factors, including mean age, HbA1C levels, duration of DM, presence of diabetic neuropathy, retinopathy, foot complications, CVD, hypertension, microvascular complications, vascular disease, nephropathy, depression, metabolic syndrome, and diuretic treatment. Our findings significantly emphasize the heightened incidence of ED among individuals within the African subgroup of the diabetic population. Notably, mean age, duration of DM, and hypertension emerge as influential contributing factors to this phenomenon.

Heterogeneity was observed in the meta-analysis of certain factors, including mean age, smoking status, and others. To explore the potential sources of heterogeneity, we conducted subgroup analyses based on various parameters. In the subgroup analysis encompassing BMI, smoking status, HbA1C, hypertension, and microvascular disease factors, our observations indicate that heterogeneity in the meta-analysis results of these factors may stem from the subgroups of region, diabetes types, and study design. Regrettably, our analyses did not reveal a significant source of heterogeneity in the results of subgroup analyses regarding mean age and duration of DM factors. In the context of conducting a meta-analysis that encompasses a substantial number of studies, it is inevitable to encounter high heterogeneity. On one hand, the vast number of studies reflects the inclusion of diverse possibilities from various sources. On the other hand, in our pursuit of incorporating a comprehensive range of risk factors to provide a broader perspective, certain quality control measures had to be relaxed, which may have introduced heterogeneity due to the inclusion of lower-quality studies under less standardized study designs. We have acknowledged and outlined the limitations of our study, which detail the reasons behind the heterogeneity. Upon thorough examination of our data, we have identified the presence of publication bias in the meta-analysis pertaining to mean age, HbA1C, and duration of DM factors. To mitigate this issue, we firstly expanded our literature search to include not only mainstream academic databases but also gray literature, unpublished studies, and conference proceedings. Then, we used the trim and filling method to validate the results, which showed that the results were still stable after applying the necessary adjustments.

Our subgroup analysis revealed a significant increase in the incidence of ED among African subgroups of the diabetic population, particularly in relation to factors such as mean age (OR: 2.38, 95% CI = 1.52 – 5.26), duration of diabetes (OR: 3.16, 95% CI = 1.41 – 7.08), and hypertension (OR: 2.23, 95% CI = 1.50 – 3.31). Several factors contribute to this increased risk of ED in African populations. Firstly, the high prevalence of chronic diseases, including CVD and hypertension, along with infectious diseases like malaria and AIDS, in specific African regions, collectively contribute to the development of ED. These diseases pose a significant burden on African regions, exacerbating the incidence of ED (76). Furthermore, it is worth noting that certain regions experience a significant economic disparity when compared to developed regions in Europe and the US. This disparity has far-reaching implications, encompassing various aspects such as the quality of medical and healthcare services, education, and food safety (77). Previous studies have established a strong correlation between these factors and the prevalence of ED (78). Finally, cultural and social contexts also play a role in the higher risk of ED among African populations. In certain African cultures, male sexual competence is considered a symbol of honor and dignity. Consequently, men may experience anxiety and stress regarding their sexual ability, which can further affect their sexual function (79).

The relationship between DM and ED has garnered significant attention in the realm of ED-related research. Conducted as a comprehensive exploration of the medical and psychosocial factors associated with erectile dysfunction, the Massachusetts Male Aging Study uncovered a significant finding: diabetic patients exhibited a threefold age-adjusted likelihood of developing ED compared to non-diabetic patients (80). In 2017, Kouidrat et al. carried out an extensive meta-analysis consisting of 145 studies. The analysis revealed prevalence rates of 37.5%, 66.3%, and 57.7% for ED in individuals with type 1, type 2, and both types of diabetes, respectively (81). Recently, a review conducted by Giuseppe Defeudis and colleagues (82) on the definition and incidence of ED in patients with DM, the influence of DM complications and treatment on ED, served as inspiration for our study. Building upon this research, we employed more objective statistical tools to delve deeper into the distinct impact of these influencing factors on ED.

Advancing age is associated with a notable decline in organ function as well as reductions in male sex hormones. Additionally, the aging process often coincides with the simultaneous presence of other risk factors for ED. There exist misconceptions suggesting that advancing age leads to diminished sexual interest and desire. However, despite a reduction in sexual activity attributable to declining physical vigor associated with aging, engagement in sexual behavior remains prevalent among older demographics (83). In an epidemiological study carried out in the UK, results indicated that as many as 84.5% of men aged 60–69 years reported participating in sexual activity, while the percentage stood at 59.3% for men aged 70–79 years (84). Our study not only provides compelling evidence for this perspective, but our subgroup analysis also reveals a noteworthy finding: populations from Africa may exhibit a heightened susceptibility to the impact of advancing age on ED (**Table 3**).

**Table 3:** Subgroup analysis of the correlation between risk factors for erectile dysfunction and diabetes mellitus.

Category of variables	No. of studies	OR(95%CI)	P	Heterogeneity	
				I^2^	P
**Mean age**	44	1.31(1.24,1.37)	<.001	94.8	<.001
Region					
** **Africa	10	2.38(1.52,5.26)	<.001	93.4	<.001
** **Asia	17	1.42(1.30,1.54)	<.001	95.0	<.001
** **Europe	5	1.96(1.56,2.47)	<.001	98.1	<.001
** **North America	9	1.14(1.06,1.21)	<.001	91.5	<.001
Kind of DM					
** **Type 1 diabetes	6	1.16(1.09,1.24)	<.001	88.7	<.001
** **Type 2 diabetes	33	1.27(1.21,1.34)	<.001	93.3	<.001
Medication for DM					
** **Yes	24	1.23(1.17,1.30)	<.001	91.6	<.001
** **No	10	1.34(1.15,1.57)	<.001	94.4	<.001
Methods of ED ascertainment					
** **IIEF	38	1.30(1.24,1.37)	<.001	94.9	<.001
** **SHIM	1	1.20(1.06,1.36)	<.001	87.0	<.001
Sample size					
** <**500	29	1.37(1.27,1.48)	<.001	91.9	<.001
** >**500	14	1.32(1.23,1.42)	<.001	97.1	<.001
Study design					
** **Cohort study	9	1.13(1.06,1.20)	<.001	93.3	<.001
** **Cross-sectional study	34	1.47(1.38,1.58)	<.001	95.0	<.001
Study population					
** **CB	8	1.28(1.17,1.41)	<.001	96.1	<.001
** **HB	35	1.35(1.27,1.43)	<.001	94.3	<.001
**BMI**	16	0.96(0.88,1.05)	0.396	88.8	<.001
Region					
** **Africa	5	0.61(0.45,0.82)	<.001	69.5	0.011
** **Asia	6	1.48(0.99,2.20)	0.053	83.2	<.001
** **Europe	2	1.03(1.00,1.07)	0.044	0.00	0.341
** **North America	2	1.41(0.69,2.85)	0.343	0.00	0.621
** **Oceania	1	1.04(1.00,1.08)	0.046	-	-
Kind of DM					
** **Type 1 diabetes	2	1.41(0.69,2.85)	0.343	0.00	0.621
** **Type 2 diabetes	14	0.96(0.87,1.05)	0.333	90.2	<.001
Medication for DM					
** **Yes	9	0.99(0.76,1.29)	0.931	89.0	<.001
** **No	4	0.97(0.88,1.07)	0.501	84.8	<.001
Sample size					
** <**500	12	1.00(0.76,1.31)	0.996	90.3	<.001
** >**500	4	1.01(0.94,1.09)	0.793	84.8	<.001
Study design					
** **Cohort study	4	1.04(1.00,1.08)	0.047	0.00	0.687
** **Cross-sectional study	12	0.95(0.84,1.07)	0.365	90.9	<.001
Study population					
** **CB	2	1.03(1.00,1.07)	0.084	0.00	0.421
** **HB	14	0.95(0.85,1.07)	0.435	89.3	<.001
**Smoking status**	16	1.32(1.18,1.47)	<.001	61.1	<.001
Region					
** **Africa	1	1.55(0.79,3.03)	0.200	-	-
** **Asia	7	1.13(0.87,1.48)	0.355	53.7	0.044
** **Europe	4	1.43(1.33,1.53)	<.001	0.00	0.400
** **North America	3	1.41(0.91,2.16)	0.121	51.7	0.126
** **Oceania	1	1.14(0.72,1.81)	0.580	-	-
Kind of DM					
** **Type 1 diabetes	3	1.68(1.26,2.25)	<.001	0.00	0.628
** **Type 2 diabetes	12	1.25(1.09,1.44)	<.001	63.4	0.002
Medication for DM					
** **Yes	9	1.31(1.07,1.61)	0.011	42.7	0.083
** **No	2	1.13(1.04,1.23)	0.004	0.00	0.971
Methods of ED ascertainment					
** **IIEF	11	1.31(1.07,1.61)	0.009	48.0	0.044
** **SHIM	1	0.84(0.52,1.36)	0.477	-	-
Sample size					
** <**500	10	1.32(1.06,1.65)	0.014	61.4	0.006
** >**500	6	1.36(1.24,1.48)	<.001	24.8	0.248
Study design					
** **Cohort study	4	1.23(0.98,1.55)	0.075	27.5	0.247
** **Cross-sectional study	19	1.34(1.19,1.52)	<.001	50.7	0.022
Study population					
** **CB	1	2.41(1.09,5.31)	0.029	-	-
** **HB	15	1.30(1.16,1.45)	<.001	61.2	<.001
**HbA1C**	26	1.44(1.28,1.62)	<.001	87.0	<.001
Region					
** **Africa	3	0.47(0.03,7.97)	0.604	96.8	<.001
** **Asia	12	1.73(1.40,2.13)	<.001	82.9	<.001
** **Europe	3	1.06(0.99,1.15)	0.105	28.8	0.245
** **North America	7	1.47(1.23,1.76)	0.079	47.0	0.079
** **Oceania	1	1.16(1.00,1.34)	0.047	-	-
Kind of DM					
** **Type 1 diabetes	5	1.34(1.11,1.63)	0.003	77.1	<.001
** **Type 2 diabetes	21	1.50(1.28,1.75)	<.001	87.7	<.001
Medication for DM					
** **Yes	14	1.68(1.38,2.04)	<.001	86.3	<.001
** **No	5	1.20(0.88,1.62)	0.248	90.9	<.001
Methods of ED ascertainment					
** **IIEF	23	1.47(1.25,1.72)	<.001	87.7	<.001
** **SHIM	1	1.12(1.01,1.25)	0.037	-	-
Sample size					
** <**500	7	1.57(1.25,1.98)	<.001	88.8	<.001
** >**500	19	1.30(1.15,1.48)	<.001	74.5	<.001
Study design					
** **Cohort study	7	1.26(1.07,1.47)	0.004	82.1	<.001
** **Cross-sectional study	20	1.58(1.30,1.92)	<.001	86.7	<.001
Study population					
** **CB	4	1.60(0.99,2.59)	0.055	78.4	0.003
** **HB	22	1.48(1.28,1.72)	<.001	84.8	<.001
**Duration of DM**	30	1.39(1.29,1.50)	<.001	95.2	<.001
Region					
** **Africa	7	3.16(1.41,7.08)	0.005	80.4	<.001
** **Asia	10	1.61(1.34,1.95)	<.001	85.5	<.001
** **Europe	7	1.34(1.13,1.59)	<.001	97.8	<.001
** **North America	5	1.06(0.94,1.18)	0.034	77.0	0.002
** **Oceania	1	1.18(1.11,1.25)	<.001	-	-
Kind of DM					
** **Type 1 diabetes	5	1.06(0.96,1.18)	0.007	87.7	<.001
** **Type 2 diabetes	21	1.30(1.19,1.42)	0.061	85.9	<.001
Medication for DM					
** **Yes	18	1.23(1.14,1.33)	<.001	91.9	<.001
** **No	6	1.21(1.08,1.37)	0.002	88.2	<.001
Sample size					
** <**500	22	1.26(1.17,1.35)	<.001	91.6	<.001
** >**500	8	1.57(1.26,1.95)	<.001	96.9	<.001
Study design					
** **Cohort study	5	1.01(0.98,1.04)	0.364	66.7	0.029
** **Cross-sectional study	25	2.01(1.68,2.41)	<.001	92.7	<.001
Study population					
** **CB	2	1.26(0.79,2.02)	0.334	78.2	0.032
** **HB	28	1.48(1.35,1.62)	<.001	95.5	<.001
**Hypertension**	28	1.74(1.52,2.00)	<.001	63.3	<.001
Region					
** **Africa	5	2.23(1.50,3.31)	<.001	0.00	0.576
** **Asia	13	1.79(1.49,2.15)	<.001	70.6	<.001
** **Europe	4	1.59(0.99,2.55)	0.053	72.4	0.013
** **North America	5	1.71(1.01,2.92)	0.047	71.5	0.007
** **Oceania	1	1.15(0.66,2.01)	0.623	-	-
Kind of DM					
** **Type 1 diabetes	2	2.17(0.45,10.42)	0.332	88.1	0.004
** **Type 2 diabetes	26	1.73(1.51,1.99)	<.001	61.6	<.001
Medication for DM					
** **Yes	19	1.75(1.44,2.12)	<.001	68.2	<.001
** **No	3	1.43(0.98,2.10)	0.066	36.2	0.208
Methods of ED ascertainment					
** **IIEF	26	1.71(1.48,1.97)	<.001	63.6	<.001
** **SHIM	2	2.38(1.19,4.74)	0.014	56.9	0.128
Sample size					
** <**500	22	1.93(1.57,2.37)	<.001	62.7	<.001
** >**500	6	1.47(1.27,1.71)	<.001	55.4	0.048
Study design					
** **Cohort study	7	1.46(0.90,2.37)	0.126	68.0	0.005
** **Cross-sectional study	21	1.78(1.54,2.05)	<.001	63.5	<.001
Study population					
** **CB	7	1.65(1.26,2.17)	<.001	74.9	<.001
** **HB	21	1.80(1.51,2.13)	<.001	59.7	<.001
**Microvascular disease**	16	2.14(1.61,2.85)	<.001	77.8	<.001
Region					
** **Africa	4	2.47(0.91,6.68)	0.076	82.6	<.001
** **Asia	10	1.81(1.48,2.21)	<.001	42.7	0.073
** **Europe	2	1.80(0.29,11.23)	0.528	91.2	<.001
Kind of DM					
** **Type 1 diabetes	1	1.43(1.09,1.88)	0.010	-	-
** **Type 2 diabetes	15	2.23(1.64,3.05)	<.001	77.3	<.001
Medication for DM					
** **Yes	15	2.23(1.64,3.05)	<.001	77.3	<.001
** **No	1	1.43(1.09,1.88)	0.01	-	-
Sample size					
** <**500	12	2.39(1.63,3.49)	<.001	74.8	<.001
** >**500	4	1.50(1.28,1.77)	<.001	0.00	0.405
Study design					
** **Cohort study	1	0.66(0.23,1.87)	0.435	-	-
** **Cross-sectional study	15	2.25(1.69,3.00)	<.001	77.9	<.001
Study population					
** **CB	2	1.17(0.55,2.48)	0.677	56.2	0.131
** **HB	14	2.36(1.72,3.24)	<.001	77.7	<.001

OR, odds ratio; CI, Confidence interval; P, P value; I2, Information Gain Ratio; CB, Community base; HB, Hospital bas; IIEF, International Index for Erectile Function.

In contrast to prior research regarding risk factors for ED (85), our study identified that BMI does not significantly contribute to ED risk. Likewise, physical activity was found to have limited efficacy in mitigating the development of ED. On one hand, it is plausible that BMI may not accurately reflect the extent of obesity in individuals, and on the other hand, managing body size and fat content may not effectively reduce ED risk in diabetic individuals without adequate glycemic control. Our findings align with this interpretation, as they underscore the significance of diabetic complications and glycemic control in relation to ED (86). Notably, the influence of smoking on ED remains considerable, underscoring its ongoing relevance. Therefore, quitting smoking represents an effective strategy for preventing and managing ED, even among individuals with DM.

Sufficient levels of androgens are crucial for erectile function. Androgens act peripherally, influencing erectile mechanisms by upholding the integrity of penile structures and regulating vasodilation in the penis (87). Two comprehensive meta-analyses, encompassing 850 diabetic men and 2000 non-diabetic individuals (88), as well as 1,822 diabetic men and 10,009 non-diabetic individuals, revealed markedly lower total testosterone levels in diabetic men compared to controls (89). This association has been linked to reduced levels of sex hormone binding globulin in individuals with DM. Our findings indicate that testosterone may not be a significant risk factor, aligning with previous reviews by Corona et al. (90), which suggest that testosterone replacement improves sexual symptoms in patients with prediabetes or newly diagnosed DM, but not in subjects with established diabetes. This phenomenon is attributed to the masking effect of diabetes-related vascular disease and neuropathy on the impact of replacement therapy.

Our observations indicate that the risk of experiencing ED is more prominently associated with diabetic complications rather than the duration of diabetes itself. These findings suggest that the duration of DM should not be perceived as the sole determinant of ED, and that the key factors contributing to heightened risk are inadequate glycemic control and the development of complications stemming from suboptimal treatment approaches. A randomized controlled study substantiates our perspective, which examined the impact of intensive glucose control on the risk of subsequent ED in 280 men with a history of diabetes ranging from 1 to 15 years and minor complications. Those initially randomized to intensive glucose control demonstrated a significantly reduced risk of ED compared to the usual care group (OR 0.33; 95% CI 0.18, 0.60) (91).

Previous animal and human studies have demonstrated that glycemic control plays a crucial role in regulating levels of systemic testosterone and Derived Factor-1 alpha. Notably, diabetic animals and humans exhibited significantly reduced levels of these two factors, whereas glycemic control effectively reversed this decline. This finding suggests that maintaining proper glycemic control mitigates the risk of ED in diabetic individuals by improving endothelial damage and enhancing protective mechanisms (92).We regret to note that only one study has investigated the outcomes of poor glycemic control as a risk factor. Consequently, we were unable to conduct a meta-analysis on this aspect. However, it is inferred that individuals with complications may be more prone to also have poor glycemic control. In individuals with diabetic retinopathy, there is an up-regulation of pro-inflammatory cytokines, which also hasten the progression of atherosclerosis. This leads to compromised blood flow to penile arterioles. Moreover, diabetic retinopathy signifies a more severe peripheral nerve complication of diabetes, undeniably exerting a detrimental impact on the erectile nerve (26). Similarly, the development of diabetic neuropathy is intricately linked to the underlying processes of microangiopathy and neurotoxicity, which manifest through a multitude of mechanisms (93). These mechanisms encompass heightened oxidative stress, accumulation of advanced glycation end products, impaired axonal transport, elevated flow through the polyol pathway, and the resulting detrimental impact on vascular nerve injury (94).

Penile erection is a complex process that involves the intricate interplay of neurovascular and psychological factors, regulating the balance between cavernous smooth muscle contraction and relaxation (95). The etiology of ED encompasses organic factors (such as neurogenic, vasogenic, steroid-induced, and drug-induced) as well as psychological factors (96). Vascular diseases, including CVD, microvascular and peripheral vascular sclerosis, and injury, are recognized as the primary organic causes of ED (97), while psychogenic ED is primarily attributed to psychological factors, social interpersonal relationships, and psychiatric diseases, all of which can exacerbate the occurrence of psychogenic ED. Hyperglycemia is frequently linked to impaired vasodilator signals, excessive smooth muscle cell contraction, and venous occlusive disorders—all of which are mechanisms contributing to ED in patients with DM (98). In addition, prolonged hyperglycemia leads to elevated oxidative stress due to factors such as inflammation, heightened production of reactive oxygen species, hyperhomocysteinemia, and reduced cellular antioxidants (99). These effects may be exacerbated by the presence of additional risk factors associated with both organic and psychological causes of ED. Our findings strongly align with this perspective, and through our meta-analysis of medical histories, we consistently observed significant impact results.

In our review of current published literature, we have identified certain risk factors that were not addressed in our article. This was due to the fact that some studies did not align with our inclusion and exclusion criteria, and others were not designed for quantitative meta-analysis. It is important to note that despite their omission from our study, these risk factors are of significance. Specifically, we would like to highlight risk factors such as abdominal obesity (100); waist circumference (101);Hypogonadism (102); Cardiovascular medications (103) encompass a range of pharmacological interventions, such as angiotensin-converting enzyme (ACE) inhibitors, calcium channel blockers, beta-blockers, and diuretics (5) that were not covered in our analysis. We recommend that future high-quality longitudinal studies with wide-ranging scopes investigate the association of these factors with ED.

A notable strength of this study lies in its status as the most comprehensive meta-analysis to date examining the risk of ED in diabetic men. Initially, our study produced noteworthy findings in African subgroups with multiple risk factors. Nevertheless, to date, there is a lack of published studies stratified by different racial/ethnic populations to ascertain the impact of diverse demographics on the incidence of ED in diabetic men. Secondly, it is important to acknowledge the significant heterogeneity observed across several of our studies. While we have attempted to address this through subgroup analysis, it is vital to recognize the limitations inherent in our interpretation of these findings. Finally, the exclusive inclusion of English literature introduces the potential for selection bias, thereby possibly limiting the ability of certain studies to conduct meta-analyses and confining them to providing solely original data.

## Conclusion

Our study indicates that in men with DM, several risk factors for ED have been identified, including mean age, HbA1C, duration of DM, diabetic neuropathy, diabetic retinopathy, diabetic foot, cardiovascular disease, hypertension, microvascular disease, vascular disease, nephropathy, depression, metabolic syndrome, and diuretic treatment. By clarifying the connection between these risk factors and ED, clinicians and scientific experts can intervene and address these risk factors, ultimately reducing the occurrence of ED and improving patient management.

## Data availability statement

The original contributions presented in the study are included in the article/**Supplementary Material**. Further inquiries can be directed to the corresponding authors.

## Ethics statement

In our current study, we solely relied on publicly accessible summary studies, and ethical approval as well as consent from participants were obtained through the original studies.

## Author contributions

DD: Conceptualization, Investigation, Methodology, Resources, Writing – original draft. AW: Investigation, Methodology, Resources, Writing – original draft. AT: Investigation, Methodology, Resources, Writing – original draft. LWT: Investigation, Methodology, Resources, Writing – original draft. AZ: Conceptualization, Funding acquisition, Supervision, Writing – review & editing. MR: Conceptualization, Funding acquisition, Supervision, Writing – review & editing.
