# Supplementary material for: Risk factors for erectile dysfunction in diabetes mellitus: a systematic review and meta-analysis
Source: Front Endocrinol (Lausanne). 2024 Apr 4;15:1368079. doi: 10.3389/fendo.2024.1368079 (PMC11024441; doi:10.3389/fendo.2024.1368079)
Supplement: Supplementary file 1 [file DataSheet_1.docx]

**Supplementary Material 1**

**Pubmed**

#1 ((((("Sexual Dysfunction, Physiological"[Mesh]) OR ("Sexual Dysfunctions, Psychological"[Mesh])) OR (sexual dysfunction)) OR (sexual function)) OR (female sexual dysfunction)) OR (FSD)

#2 (((((((((((Metabolic syndrome[MeSH]) OR (Metabolic Syndrome)) OR (Dysmetabolic Syndrome)) OR (Cardiometabolic Syndrome)) OR (Cardiovascular Syndrome, Metabolic)) OR (Metabolic X Syndrome)) OR (Syndrome X)) OR (deadly quartet)) OR (insulin resistance syndrome)) OR (Insulin Resistance Syndrome X)) OR (Reaven's Syndrome)) OR (metS)

#3 NOT ("case reports"[Publication Type] OR "comment"[Publication Type] OR "editorial"[Publication Type] OR "letter"[Publication Type] OR animal)

#1 AND #2 NOT #3

#4 (((((("Sexual Dysfunction, Physiological"[Mesh]) OR ("Sexual Dysfunctions, Psychological"[Mesh])) OR (sexual dysfunction)) OR (sexual function)) OR (female sexual dysfunction)) OR (FSD)) AND ((((((((((((Metabolic syndrome[MeSH]) OR (Metabolic Syndrome)) OR (Dysmetabolic Syndrome)) OR (Cardiometabolic Syndrome)) OR (Cardiovascular Syndrome, Metabolic)) OR (Metabolic X Syndrome)) OR (Syndrome X)) OR (deadly quartet)) OR (insulin resistance syndrome)) OR (Insulin Resistance Syndrome X)) OR (Reaven's Syndrome)) OR (metS)) NOT ("case reports"[Publication Type] OR "comment"[Publication Type] OR "editorial"[Publication Type] OR "letter"[Publication Type] OR animal)

**Scopus**

#1 "Metabolic Syndrome" OR " Dysmetabolic Syndrome" OR "Cardiometabolic Syndrome" OR "Cardiovascular Syndrome, Metabolic" OR "Metabolic X Syndrome" OR "Syndrome X" OR "insulin resistance syndrome" OR "Insulin Resistance Syndrome X" OR "Reaven's Syndrome" OR "metS"

#2 TITLE-ABS ( sexual AND dysfunction ) OR TITLE-ABS ( sexual AND function ) OR TITLE-ABS ( female AND sexual AND dysfunction )

#3 #1 AND #2 AND ( LIMIT-TO ( DOCTYPE , "ar" ) ) AND ( LIMIT-TO ( LANGUAGE , "English" ) ) AND ( LIMIT-TO ( SRCTYPE , "j" ) )

**Embase**

#1 'metabolic syndrome X' OR 'Dysmetabolic Syndrome' OR 'Metabolic X Syndrome' OR 'syndrome X'

#2 'sexual dysfunction' OR 'female sexual dysfunction' OR 'sexual function'

#3 #1 AND #2 AND [humans]/lim AND [english]/lim AND [embase]/lim

**Cochrane Library**

**#1** "Metabolic Syndrome" OR " Dysmetabolic Syndrome" OR "Cardiometabolic Syndrome" OR "Cardiovascular Syndrome, Metabolic" OR "Metabolic X Syndrome" OR "Syndrome X" OR "insulin resistance syndrome" OR "Insulin Resistance Syndrome X" OR "Reaven's Syndrome" OR "metS"

#2 'sexual dysfunction' OR 'female sexual dysfunction' OR 'sexual function'

#3 #1 AND #2

**
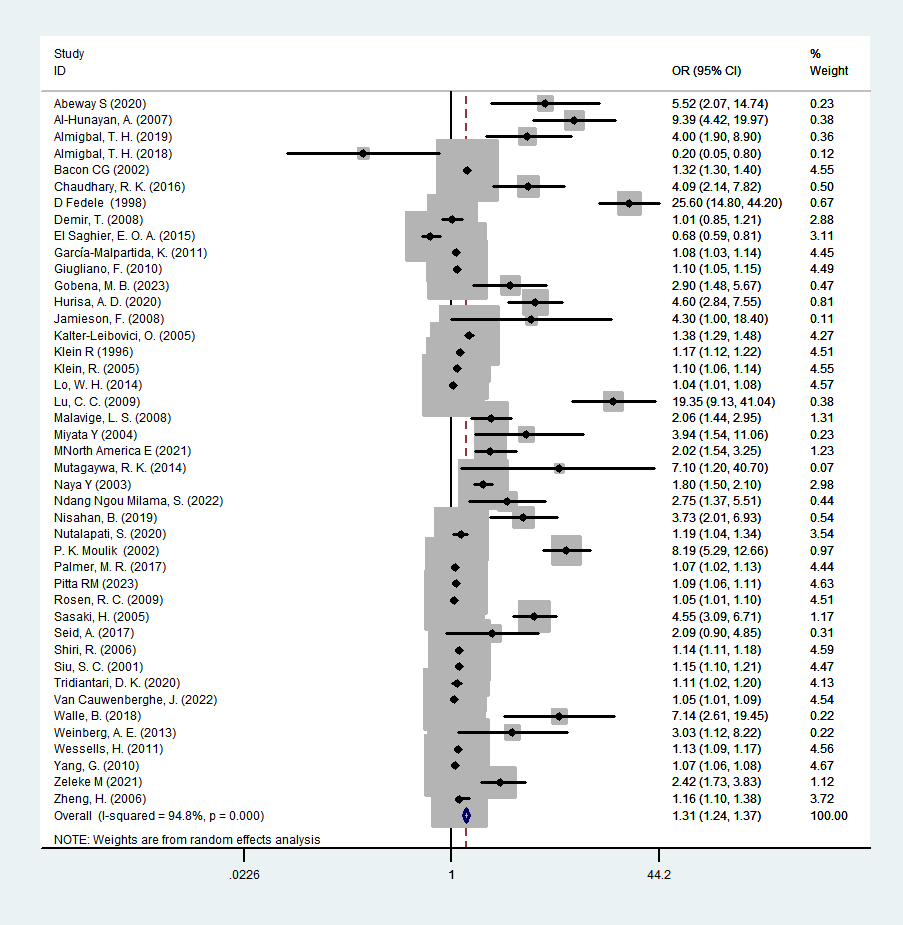
**

**Supplementary Figure 1.**  Forest plot of the association between Mean age and Erectile Dysfunction in Diabetes Mellitus.

**
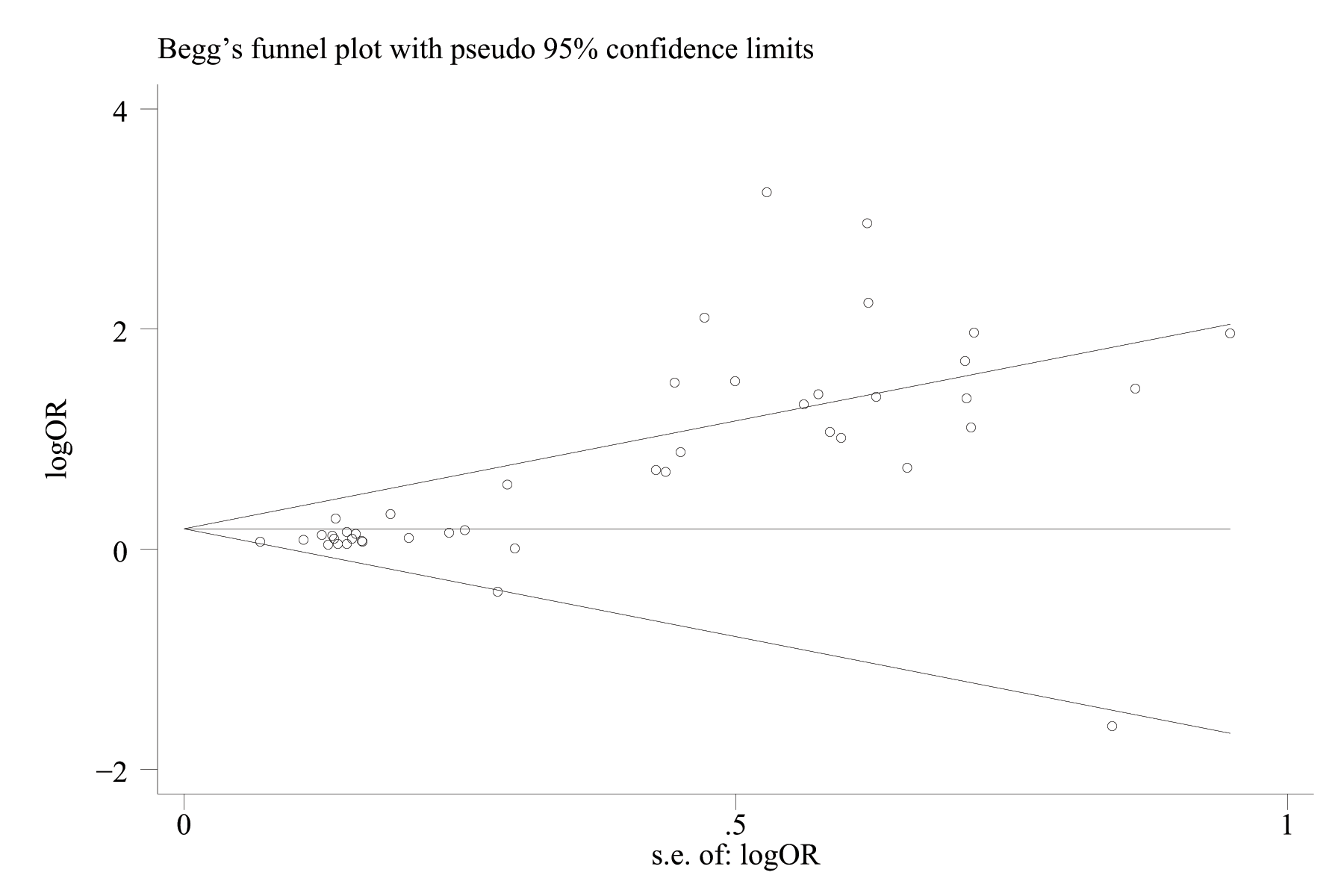
**

**Supplementary Figure 2.** Begg’s funnel plot for publication bias of association between Mean age and Erectile Dysfunction in Diabetes Mellitus.


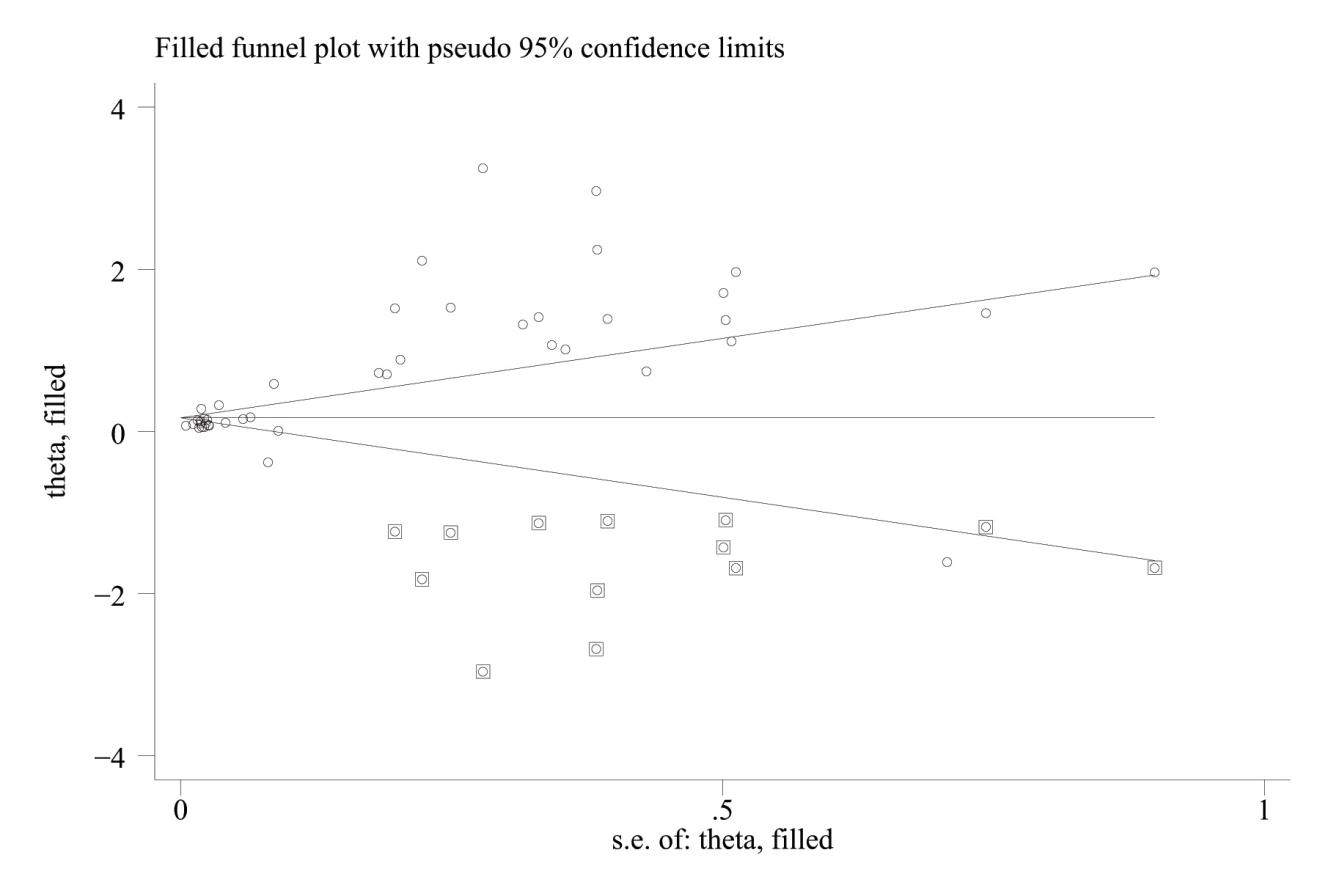


**Supplementary Figure 3.** Begg’s funnel plot for publication bias of association between Mean age and Erectile Dysfunction in Diabetes Mellitus.(trim and filling method)

**
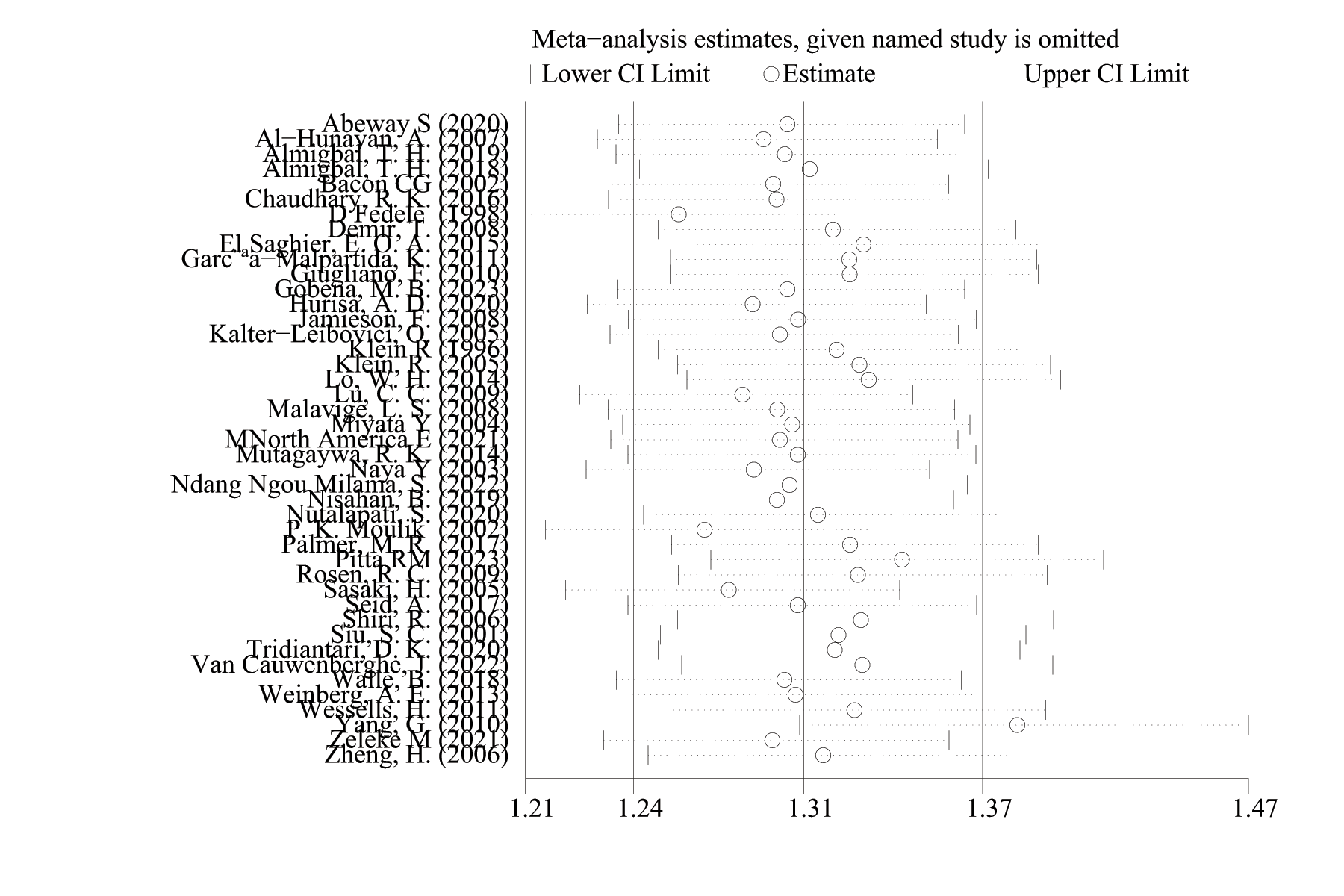
**

**Supplementary Figure 4.** Sensitivity analysis of the enrolled studies for associations between Mean age and Erectile Dysfunction in Diabetes Mellitus.

**
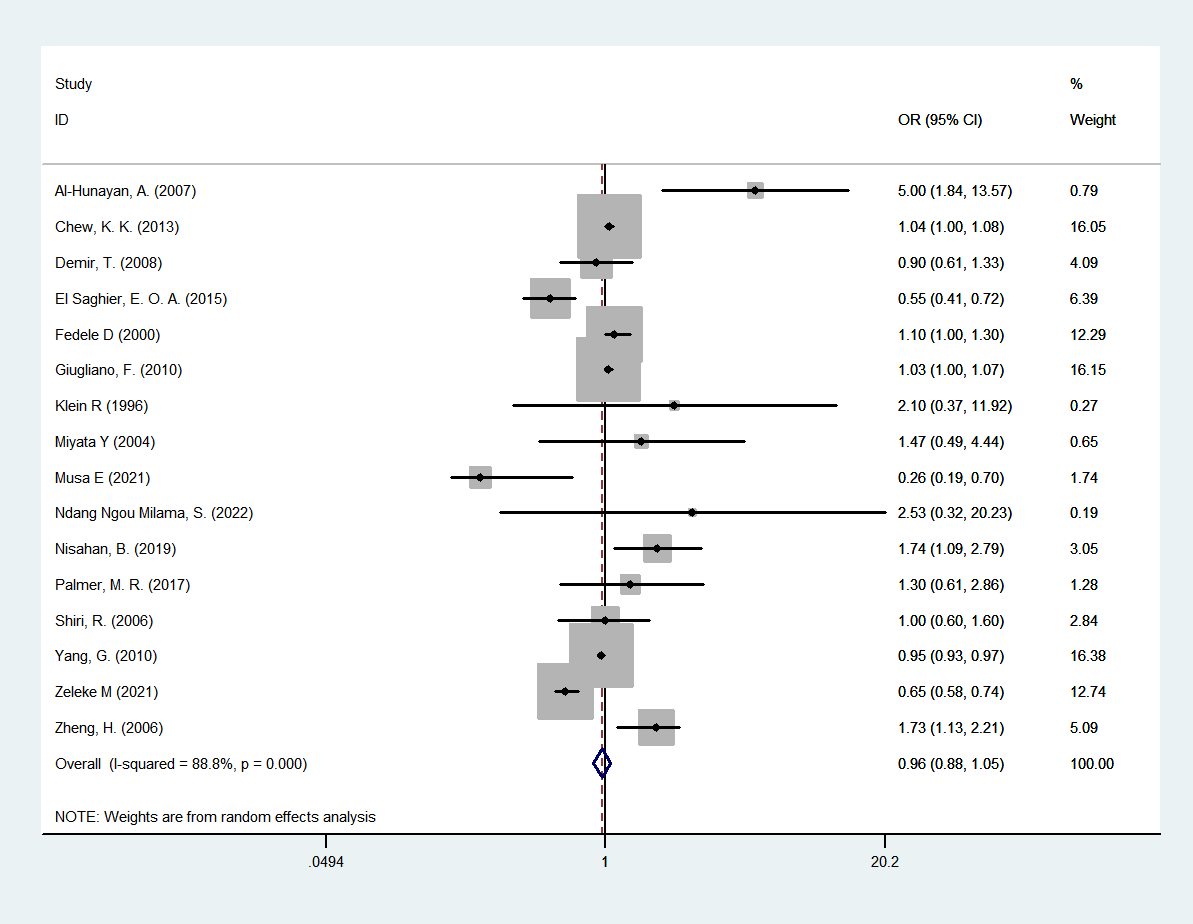
**

**Supplementary Figure 5.**  Forest plot of the association between BMI and Erectile Dysfunction in Diabetes Mellitus.

**
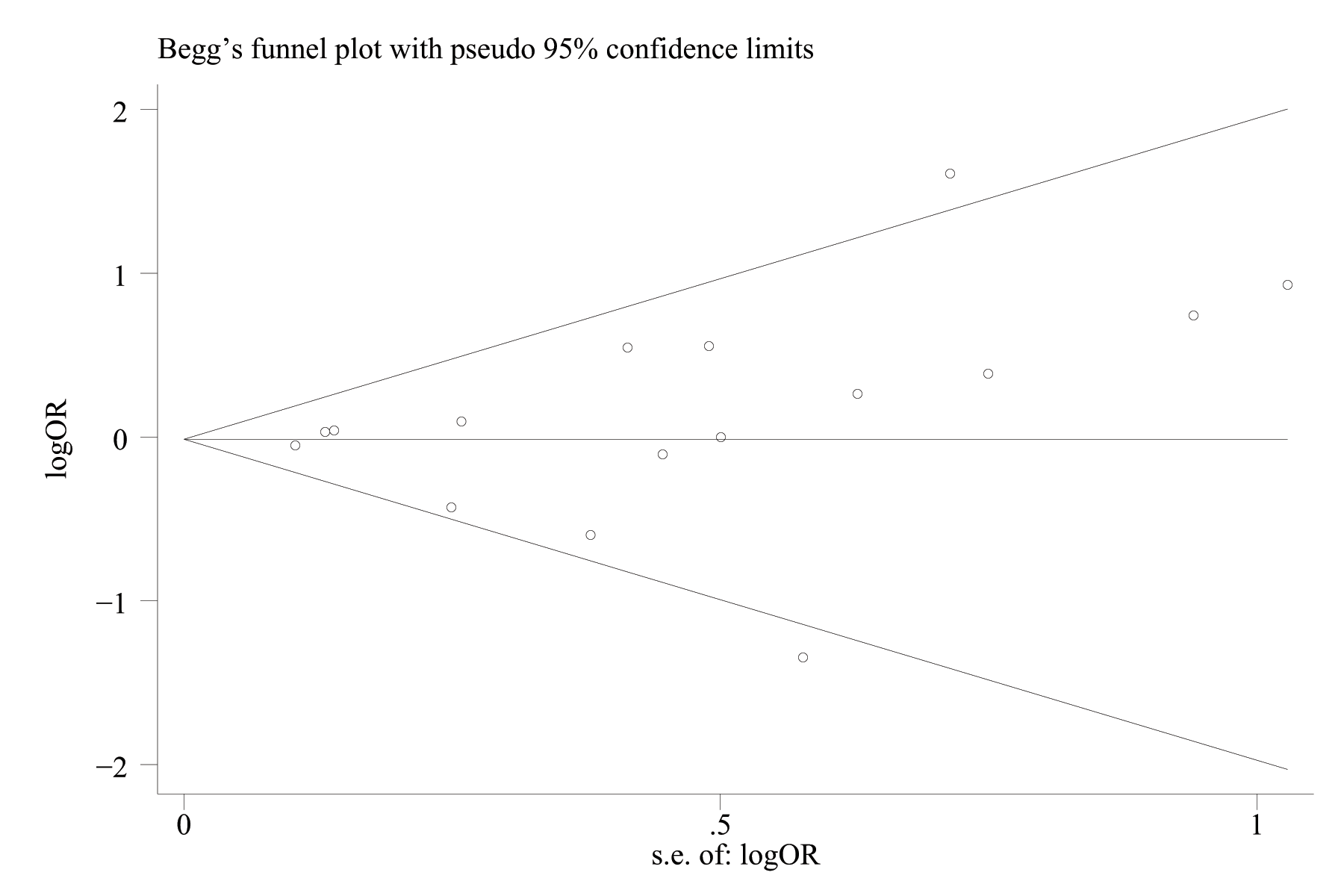
**

**Supplementary Figure 6.** Begg’s funnel plot for publication bias of association between BMI and Erectile Dysfunction in Diabetes Mellitus.


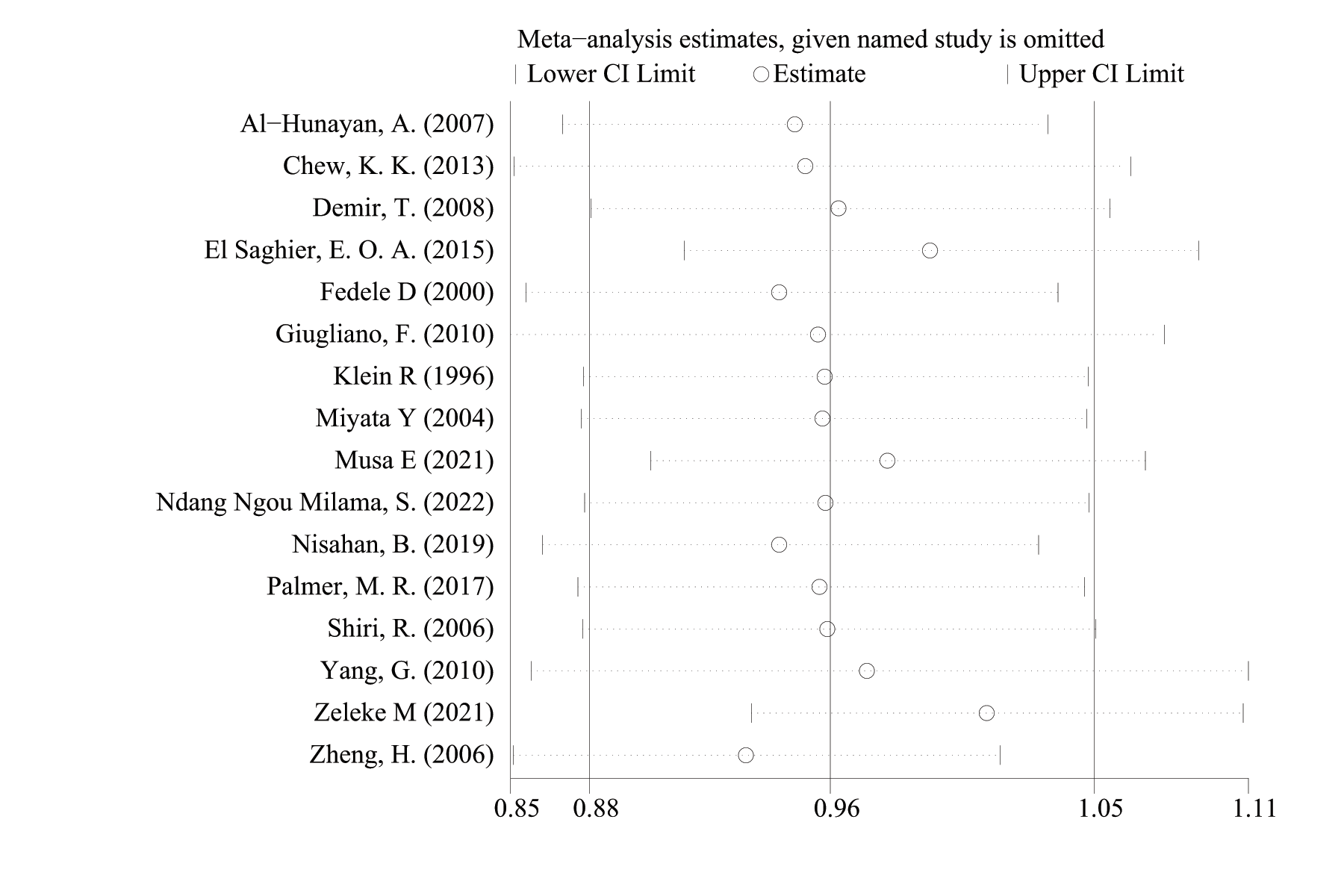

**Supplementary Figure 7.** Sensitivity analysis of the enrolled studies for associations between BMI and Erectile Dysfunction in Diabetes Mellitus.


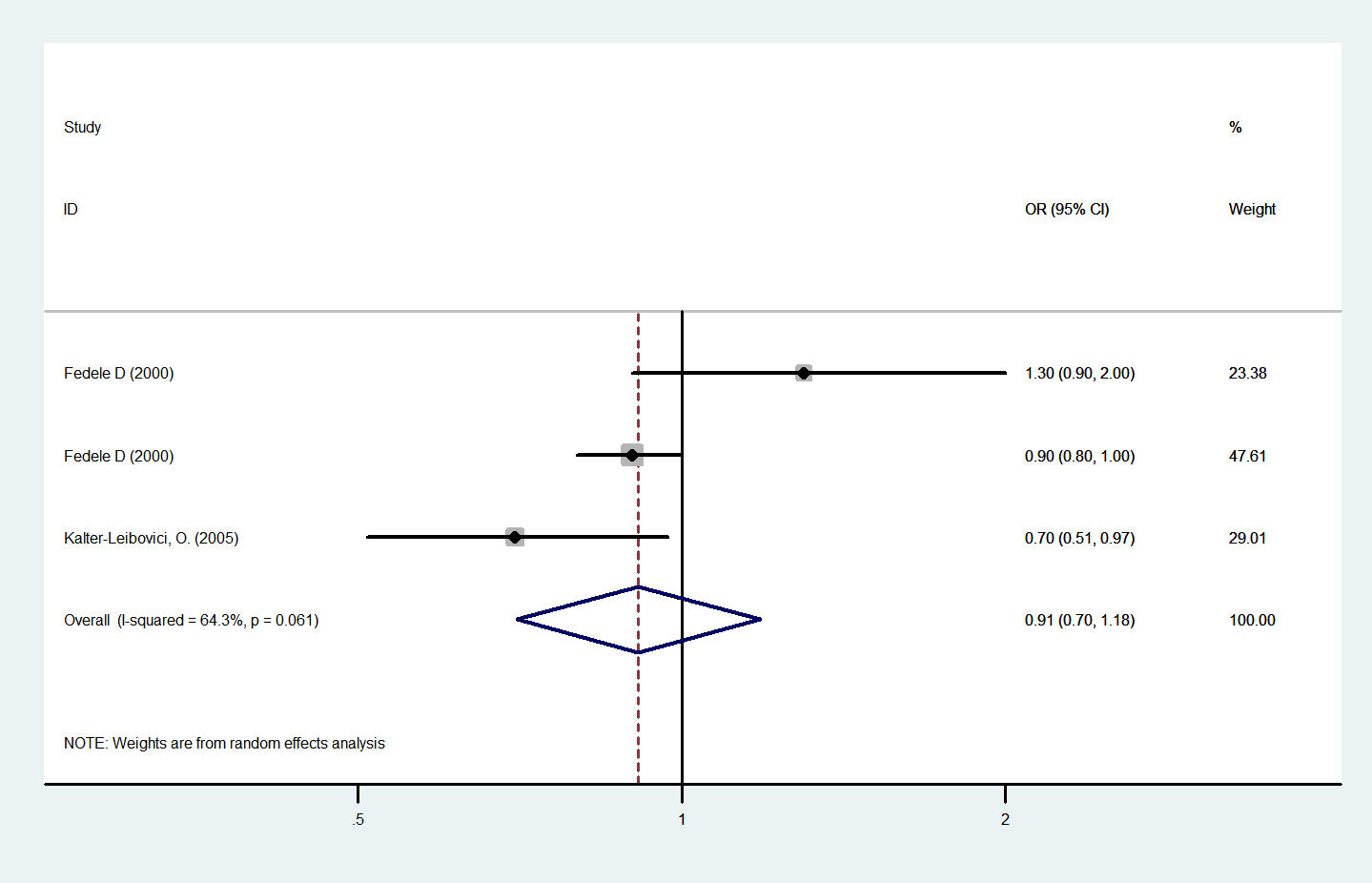


**Supplementary Figure 8.**  Forest plot of the association between Alcohol consumption and Erectile Dysfunction in Diabetes Mellitus.

**
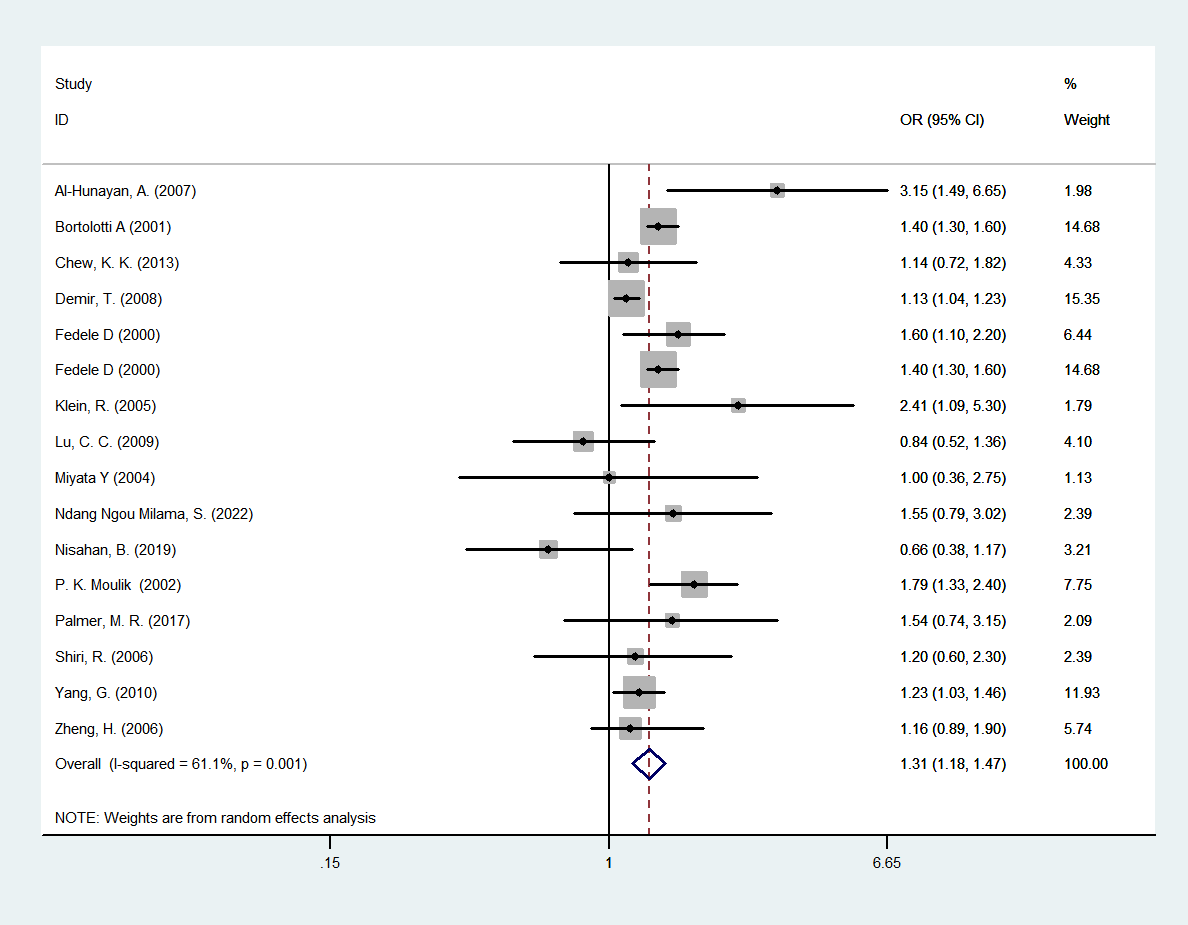
**

**Supplementary Figure 9.**  Forest plot of the association between Smoking status and Erectile Dysfunction in Diabetes Mellitus.


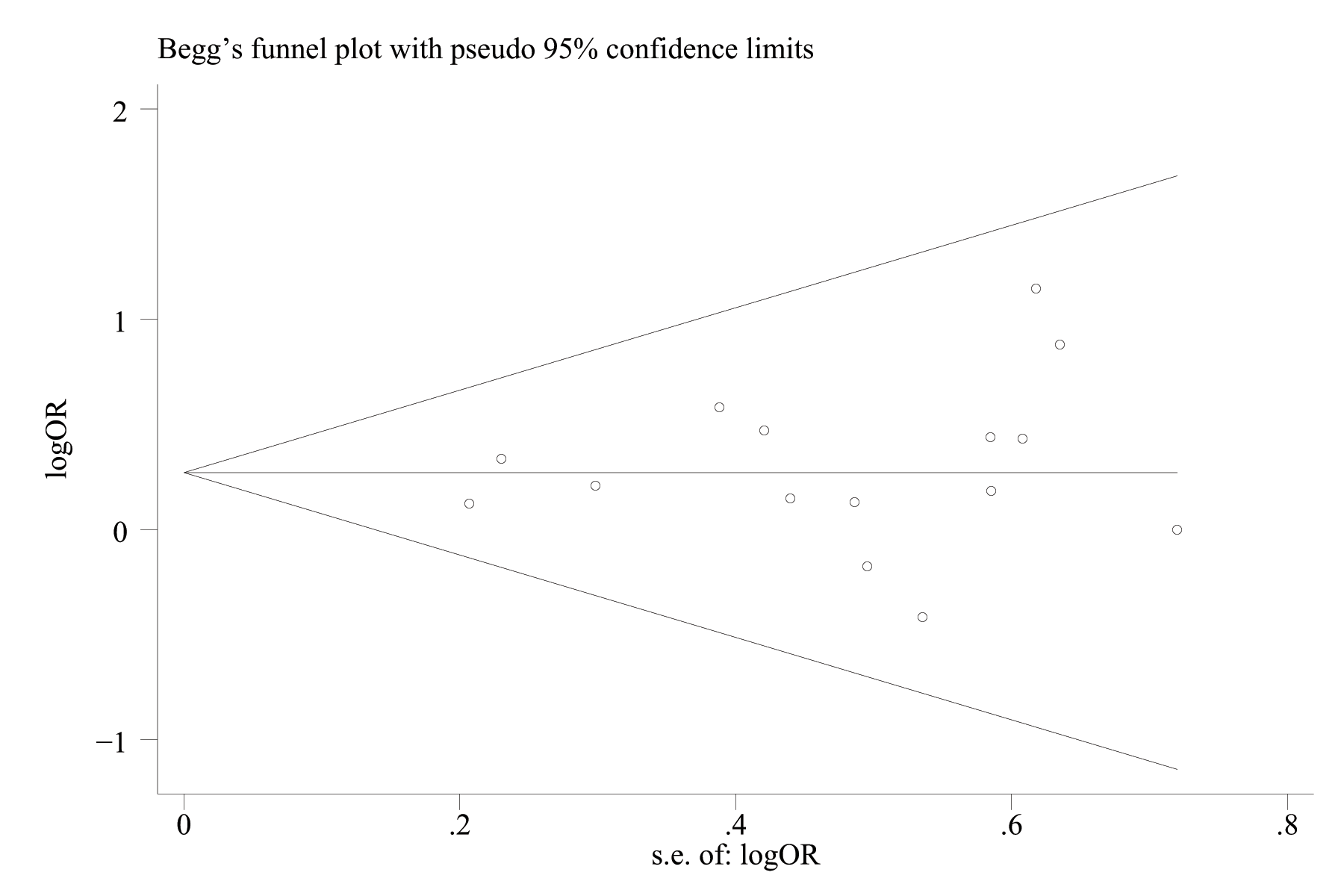


**Supplementary Figure 10.** Begg’s funnel plot for publication bias of association between Smoking status and Erectile Dysfunction in Diabetes Mellitus.


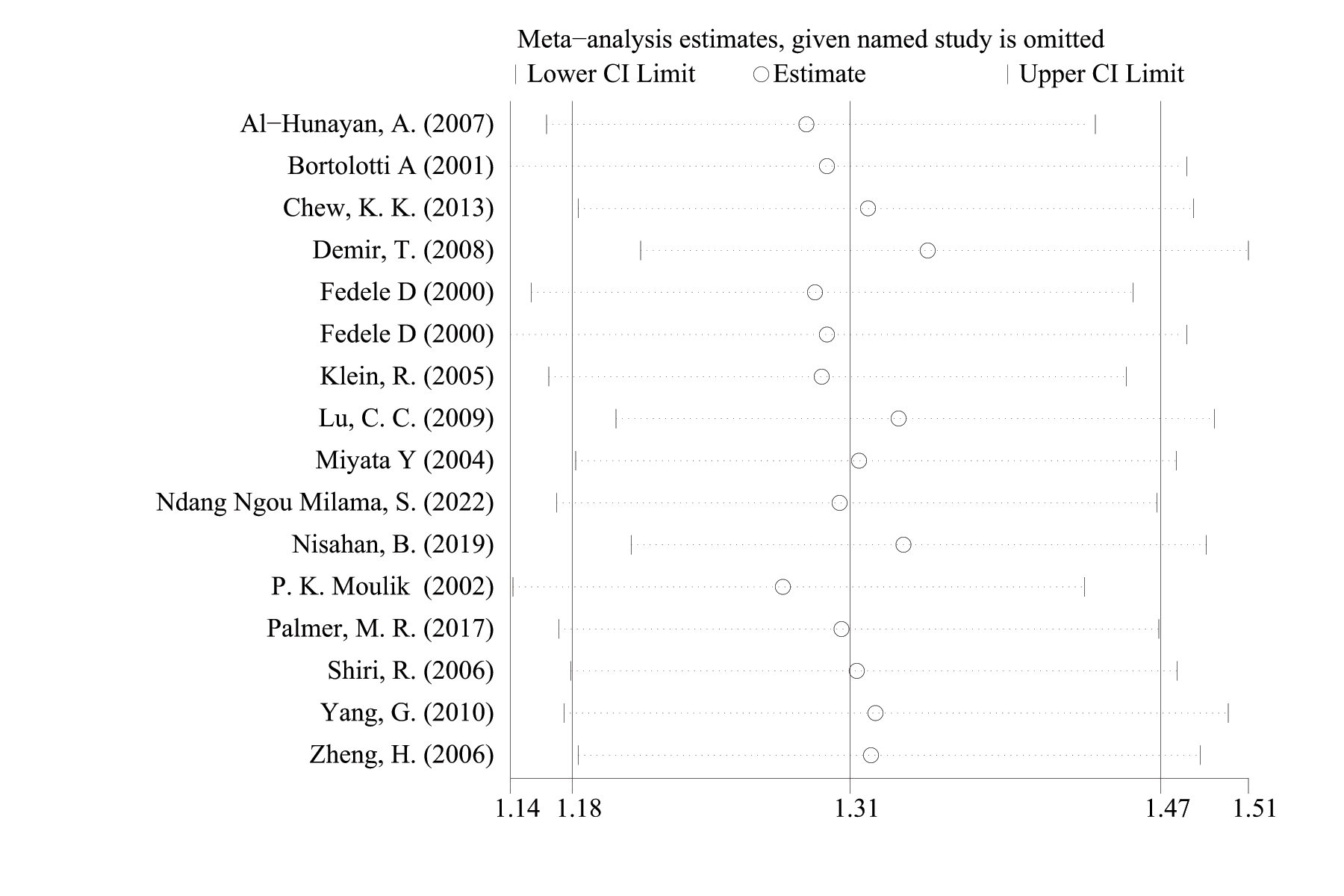


**Supplementary Figure 11.** Sensitivity analysis of the enrolled studies for associations between Smoking status and Erectile Dysfunction in Diabetes Mellitus.


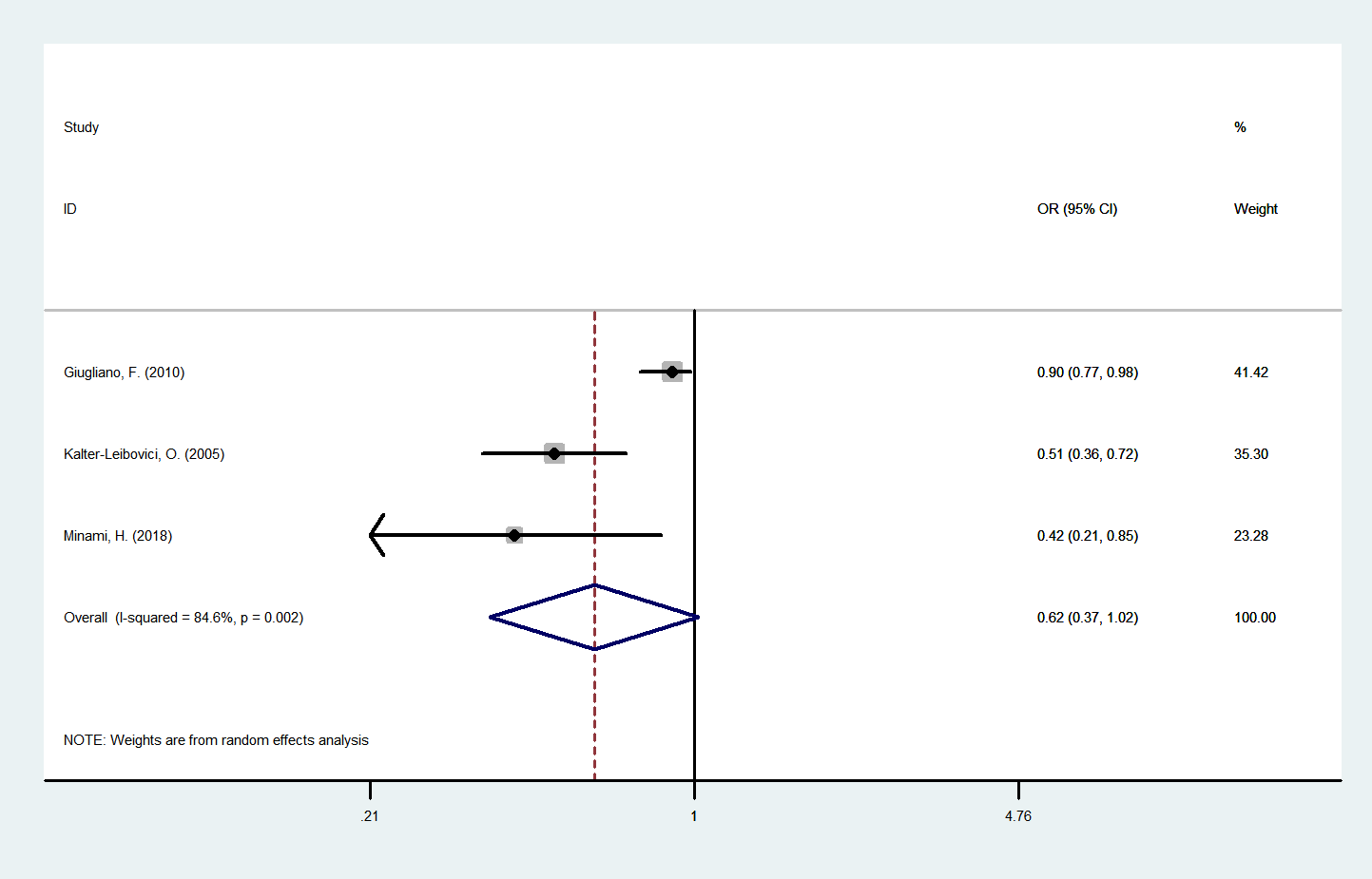


**Supplementary Figure 12.**  Forest plot of the association between Physical activity and Erectile Dysfunction in Diabetes Mellitus.


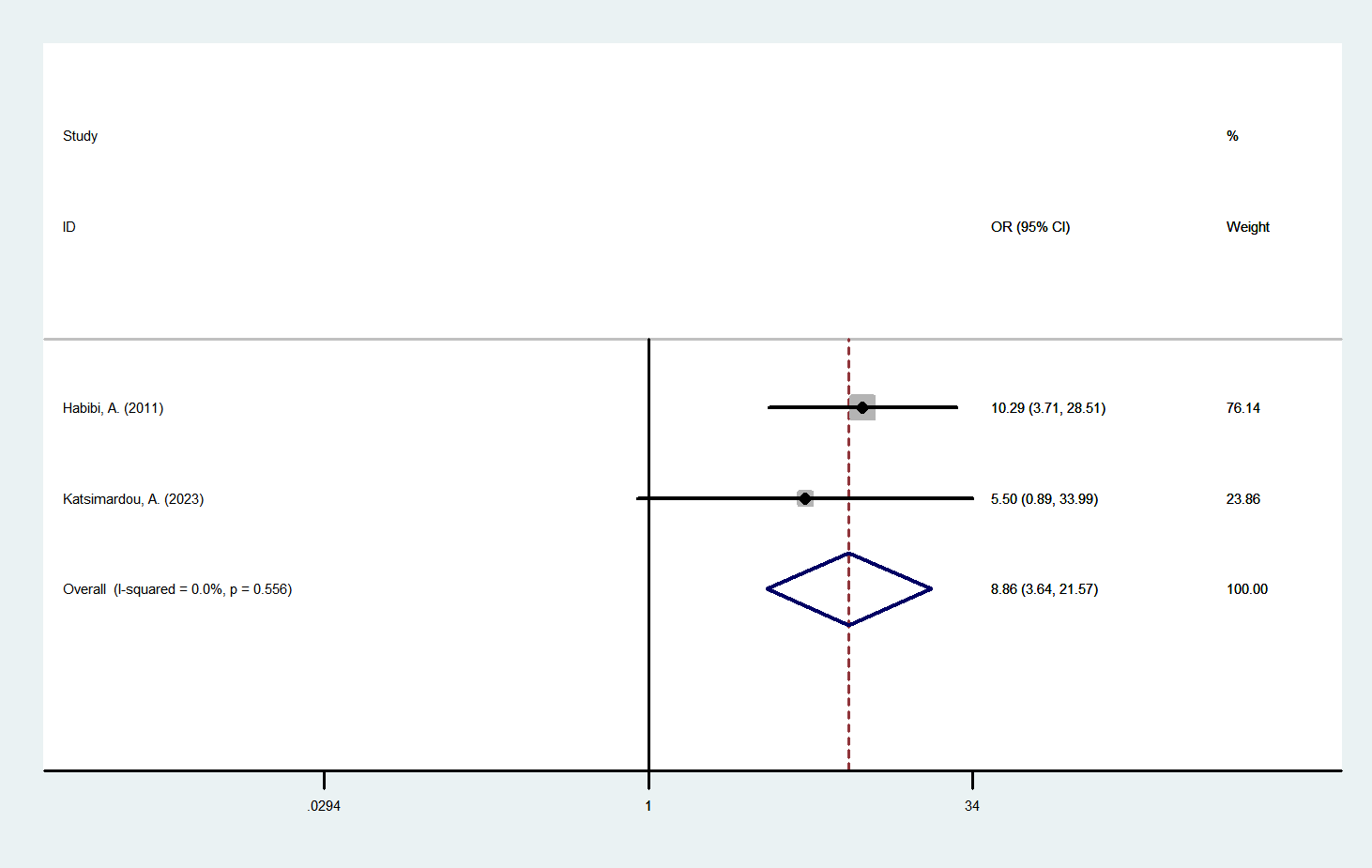


**Supplementary Figure 13.**  Forest plot of the association between Low HDL cholesterol and Erectile Dysfunction in Diabetes Mellitus.


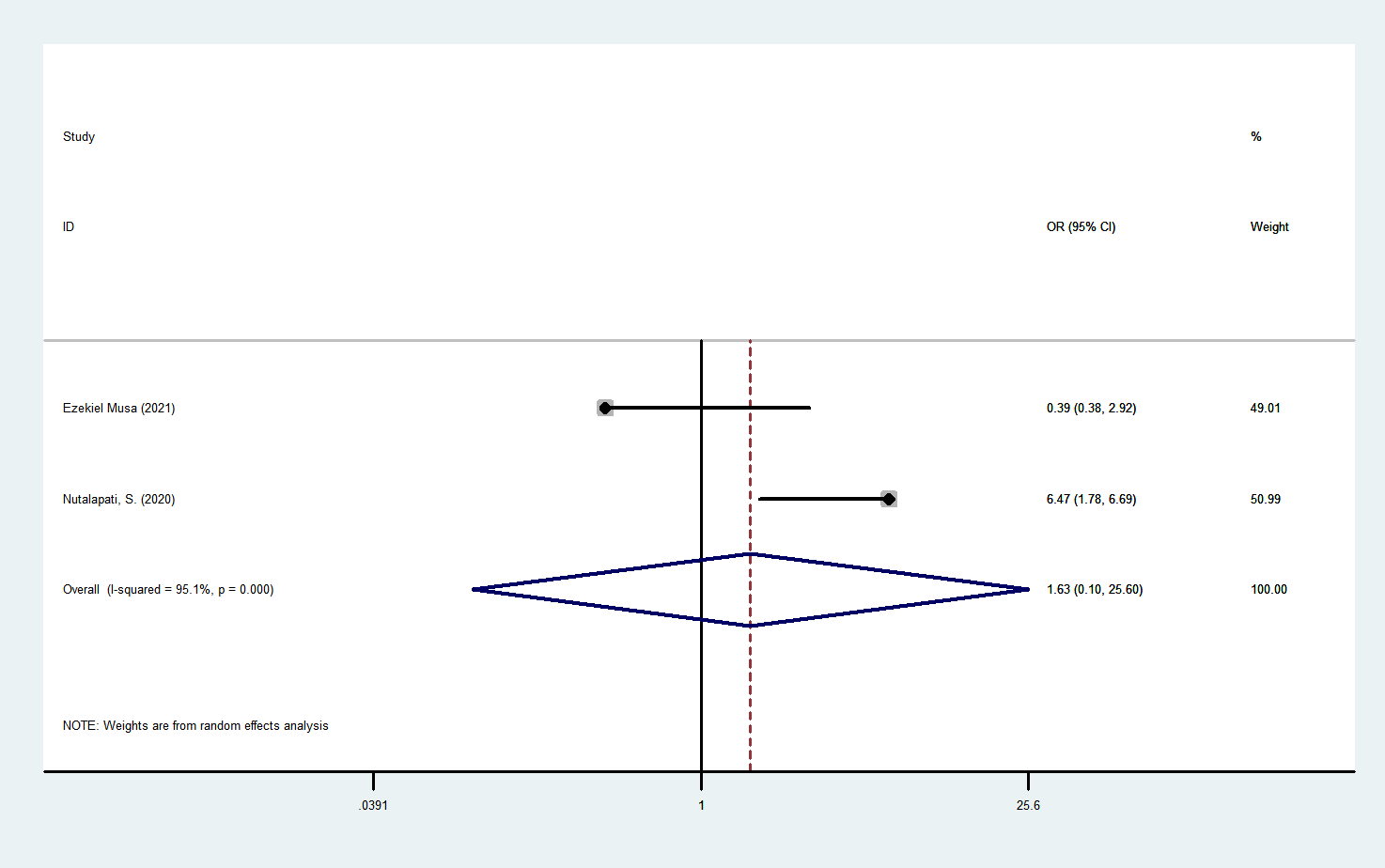


**Supplementary Figure 14.**  Forest plot of the association between Testosterone and Erectile Dysfunction in Diabetes Mellitus.


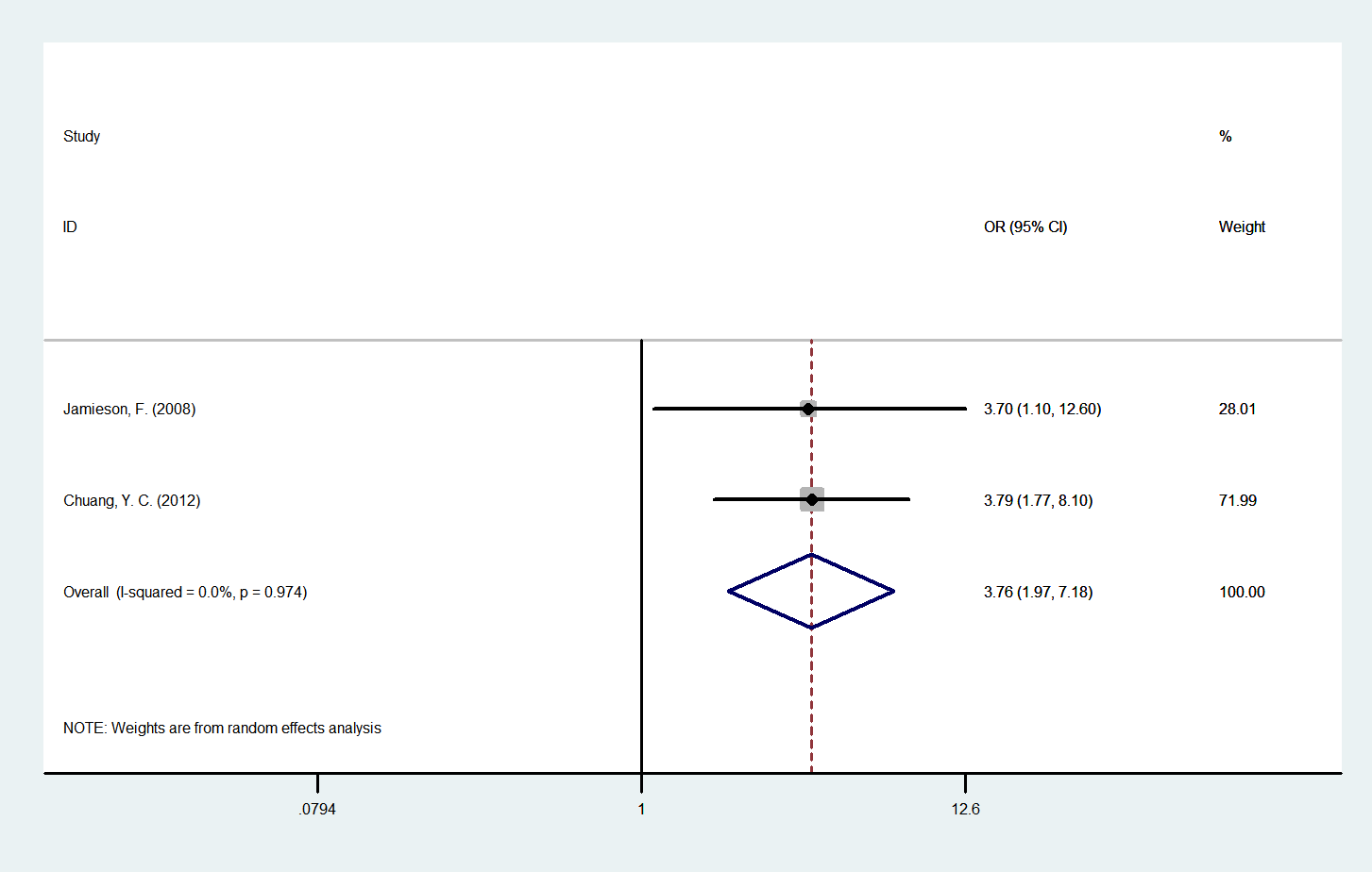


**Supplementary Figure 15.**  Forest plot of the association between Microalbuminuria and Erectile Dysfunction in Diabetes Mellitus.


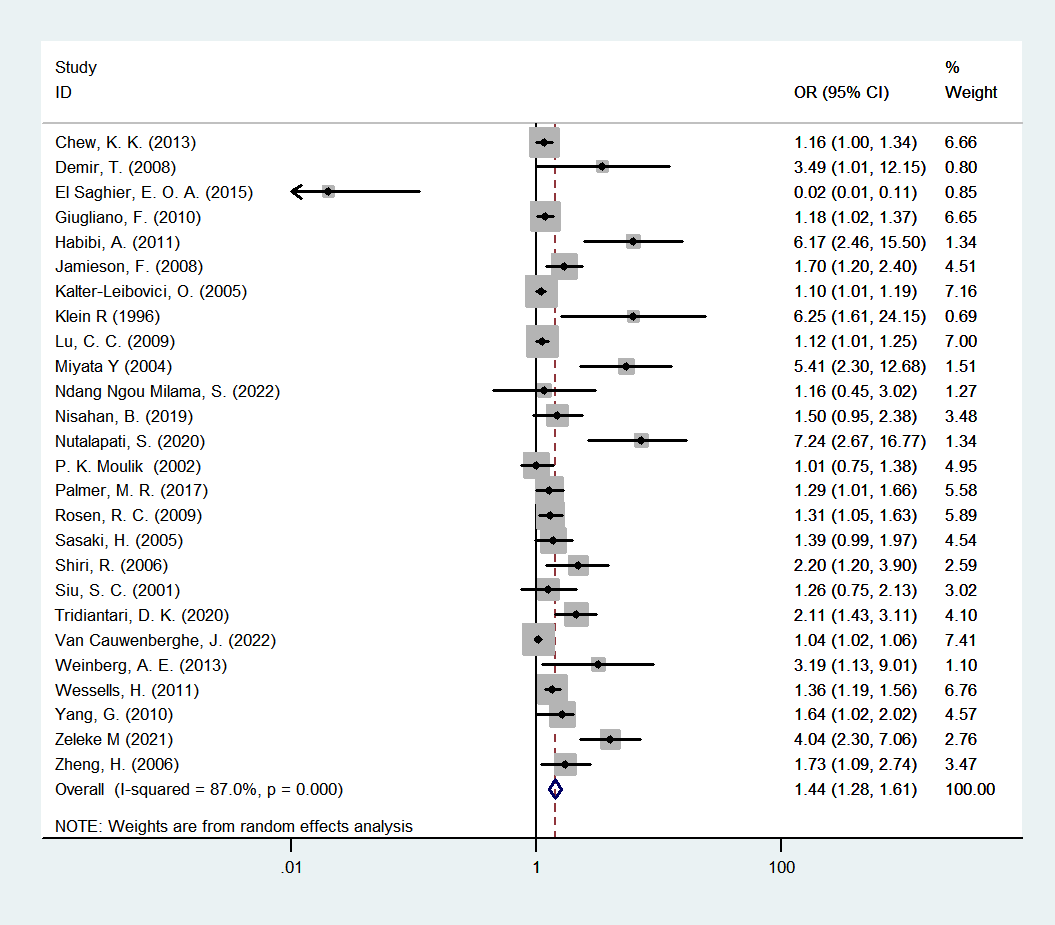


**Supplementary Figure 16.**  Forest plot of the association between HbA1C and Erectile Dysfunction in Diabetes Mellitus.


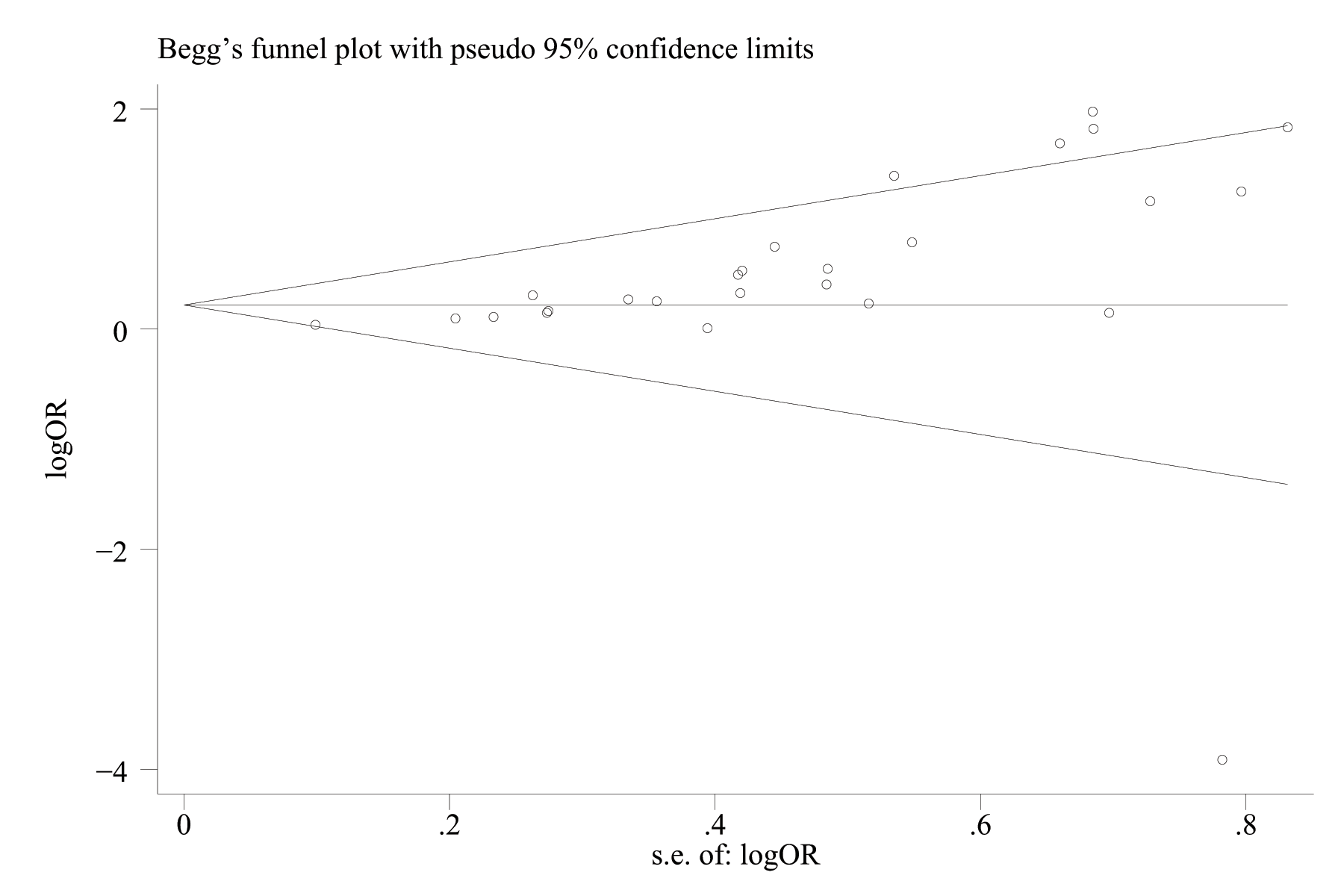


**Supplementary Figure 17.** Begg’s funnel plot for publication bias of association between HbA1C and Erectile Dysfunction in Diabetes Mellitus.


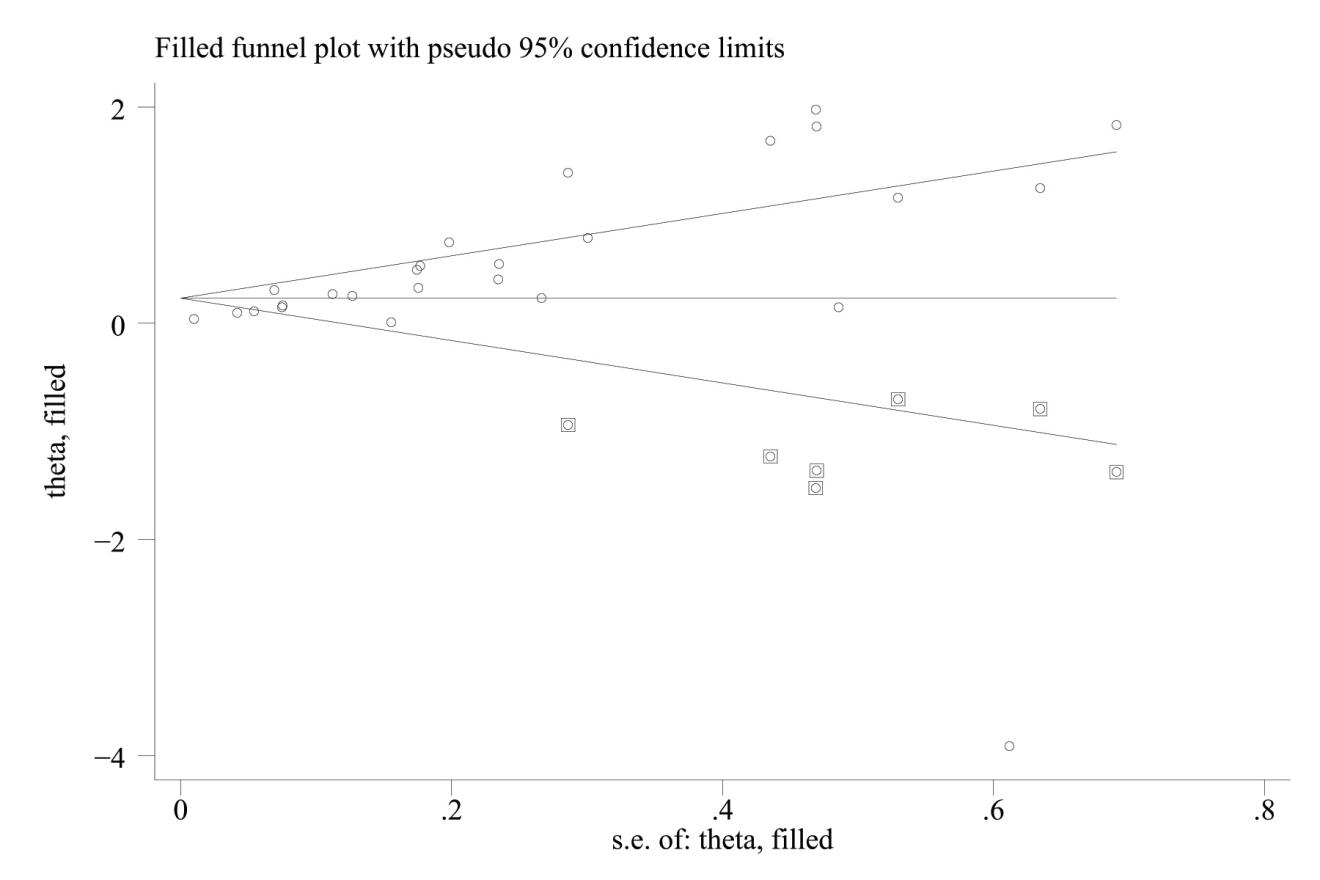


**Supplementary Figure 18.** Begg’s funnel plot for publication bias of association between HbA1C and Erectile Dysfunction in Diabetes Mellitus(trim and filling method).


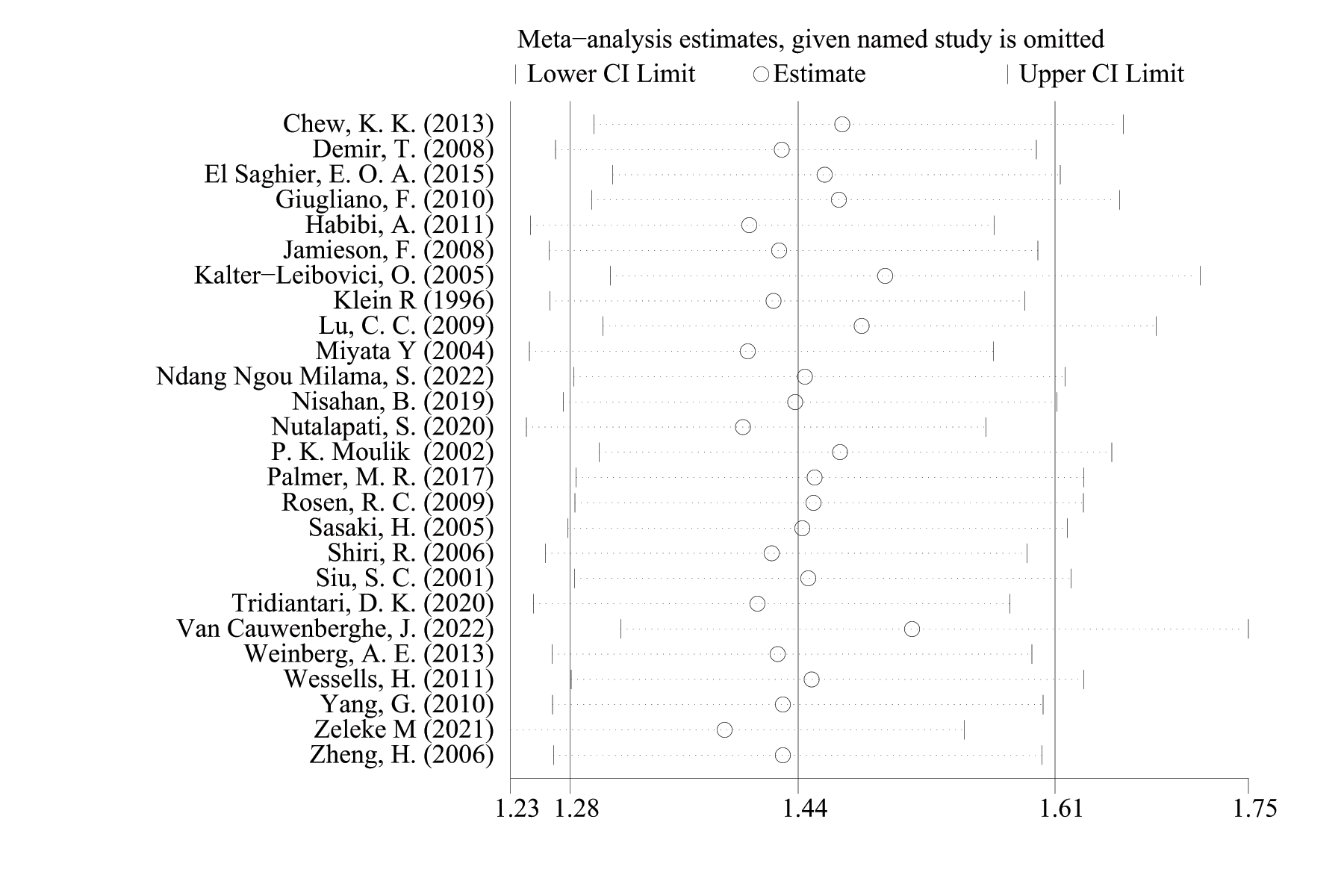


**Supplementary Figure 19.** Sensitivity analysis of the enrolled studies for associations between HbA1C and Erectile Dysfunction in Diabetes Mellitus.


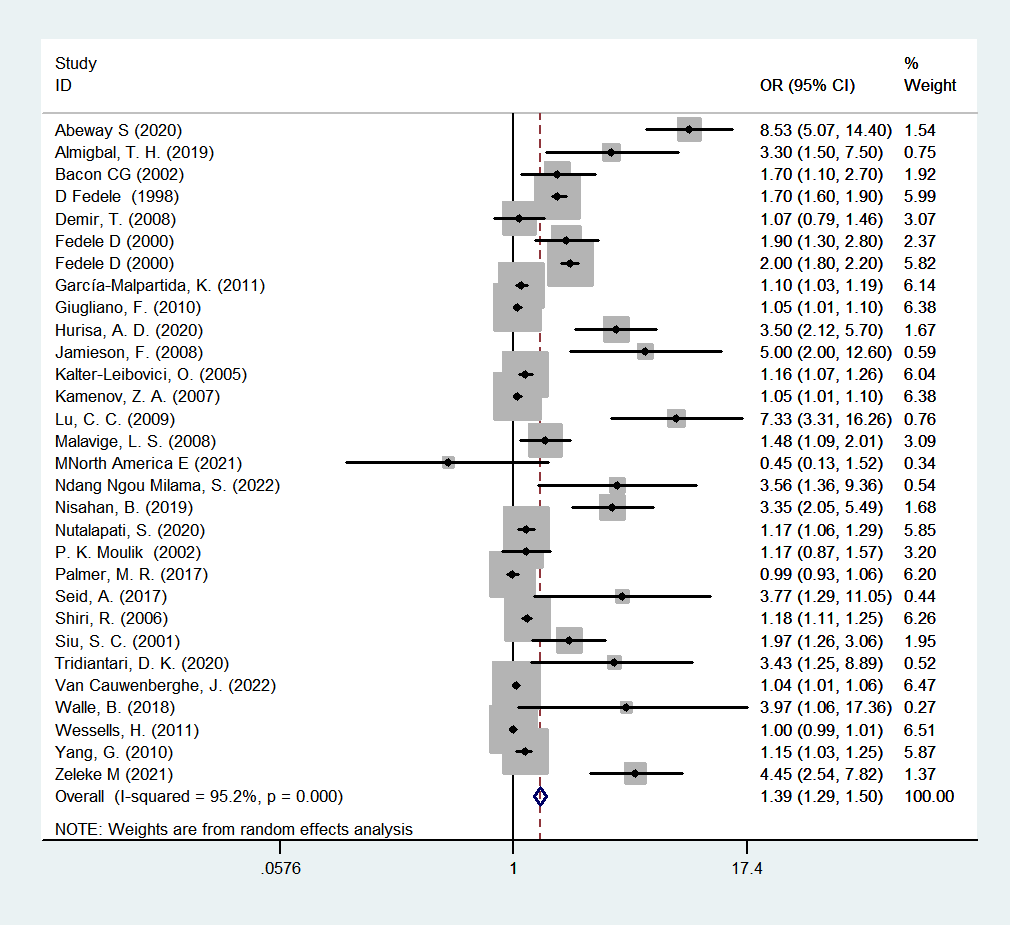


**Supplementary Figure 20.**  Forest plot of the association between Duration of DM and Erectile Dysfunction in Diabetes Mellitus.


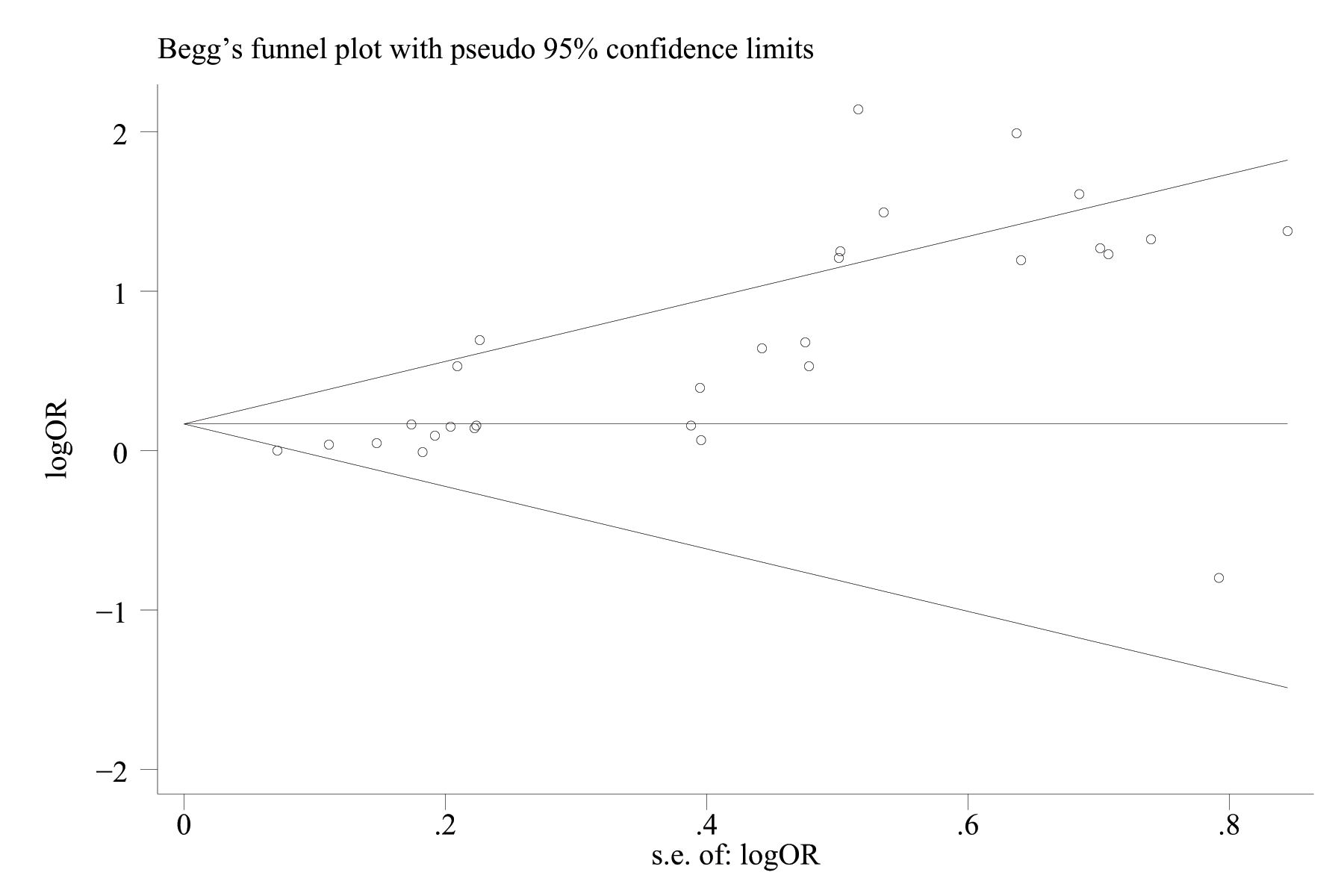


**Supplementary Figure 21.** Begg’s funnel plot for publication bias of association between Duration of DM and Erectile Dysfunction in Diabetes Mellitus.


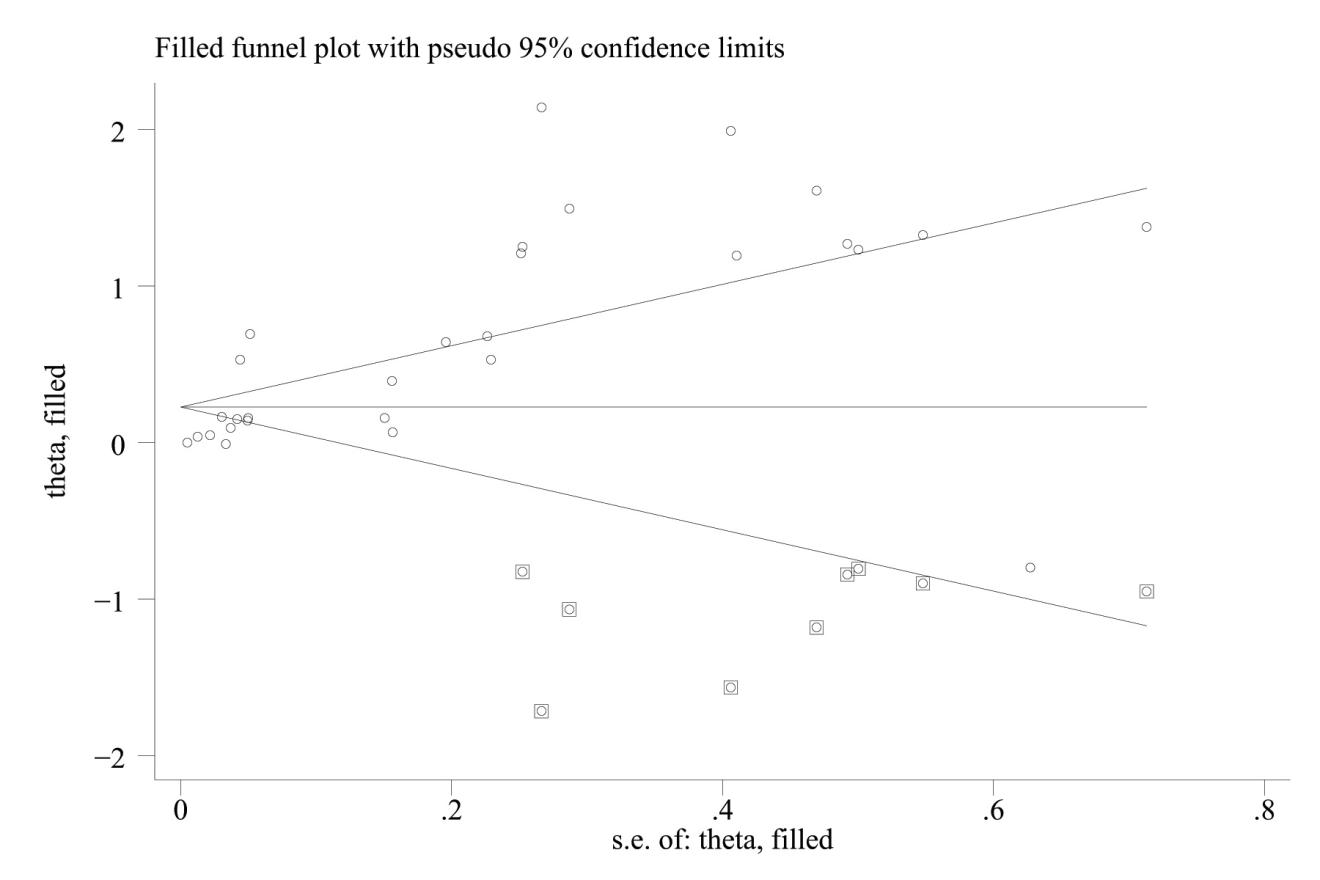


**Supplementary Figure 22.** Begg’s funnel plot for publication bias of association between Duration of DM and Erectile Dysfunction in Diabetes Mellitus(trim and filling method).


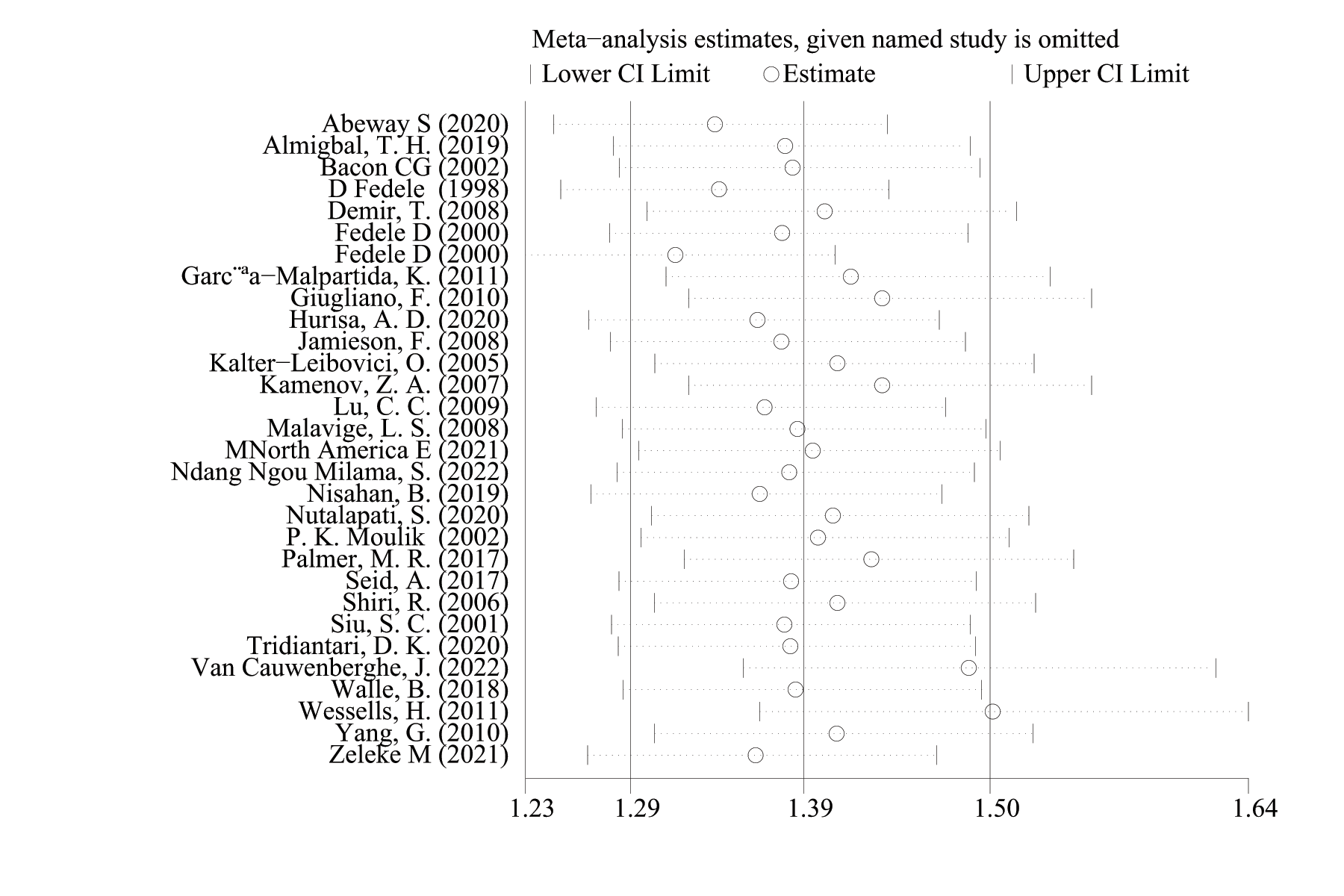


**Supplementary Figure 23.** Sensitivity analysis of the enrolled studies for associations between Duration of DM and Erectile Dysfunction in Diabetes Mellitus.


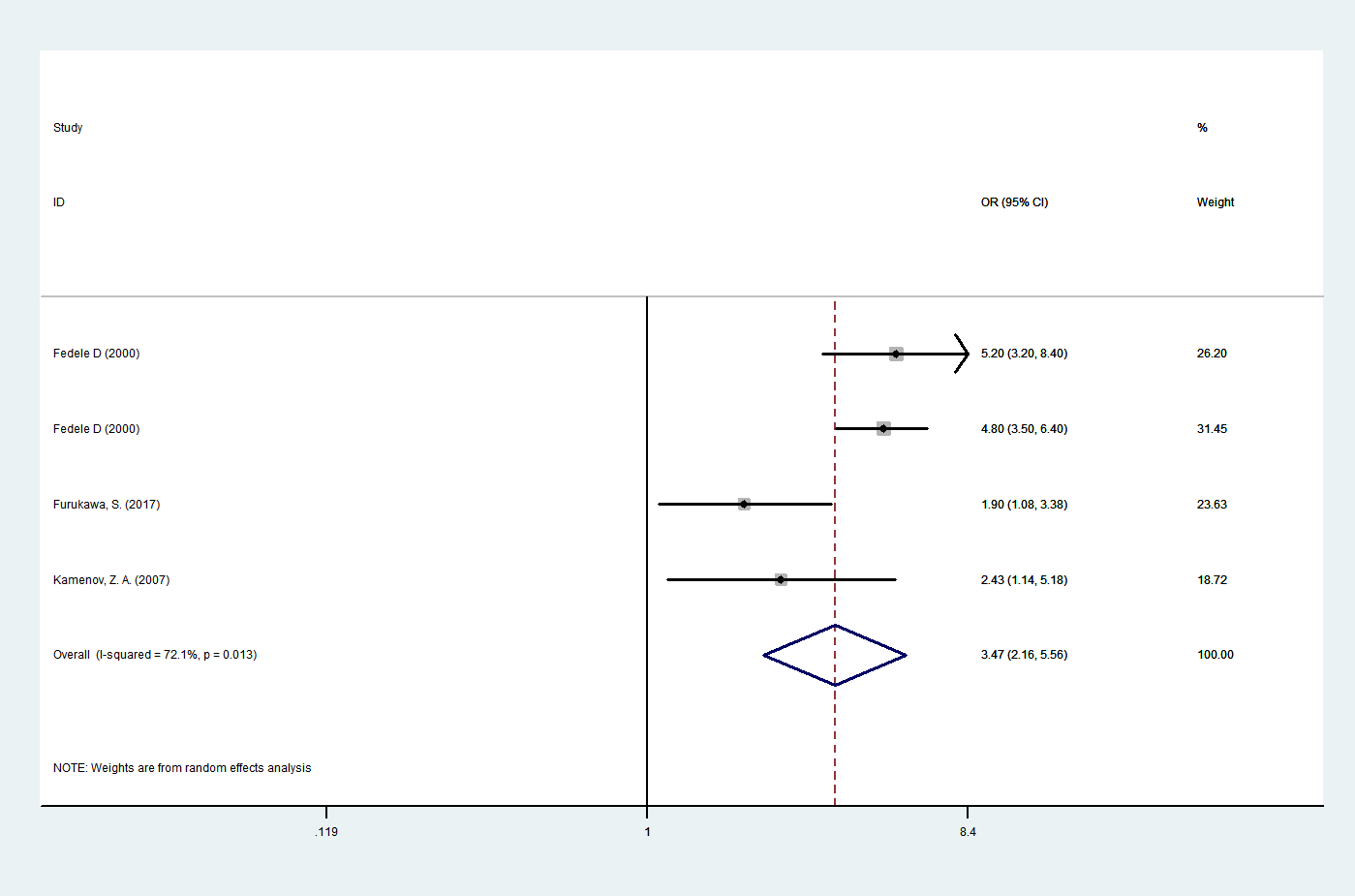


**Supplementary Figure 24.**  Forest plot of the association between Diabetic neuropathy and Erectile Dysfunction in Diabetes Mellitus.


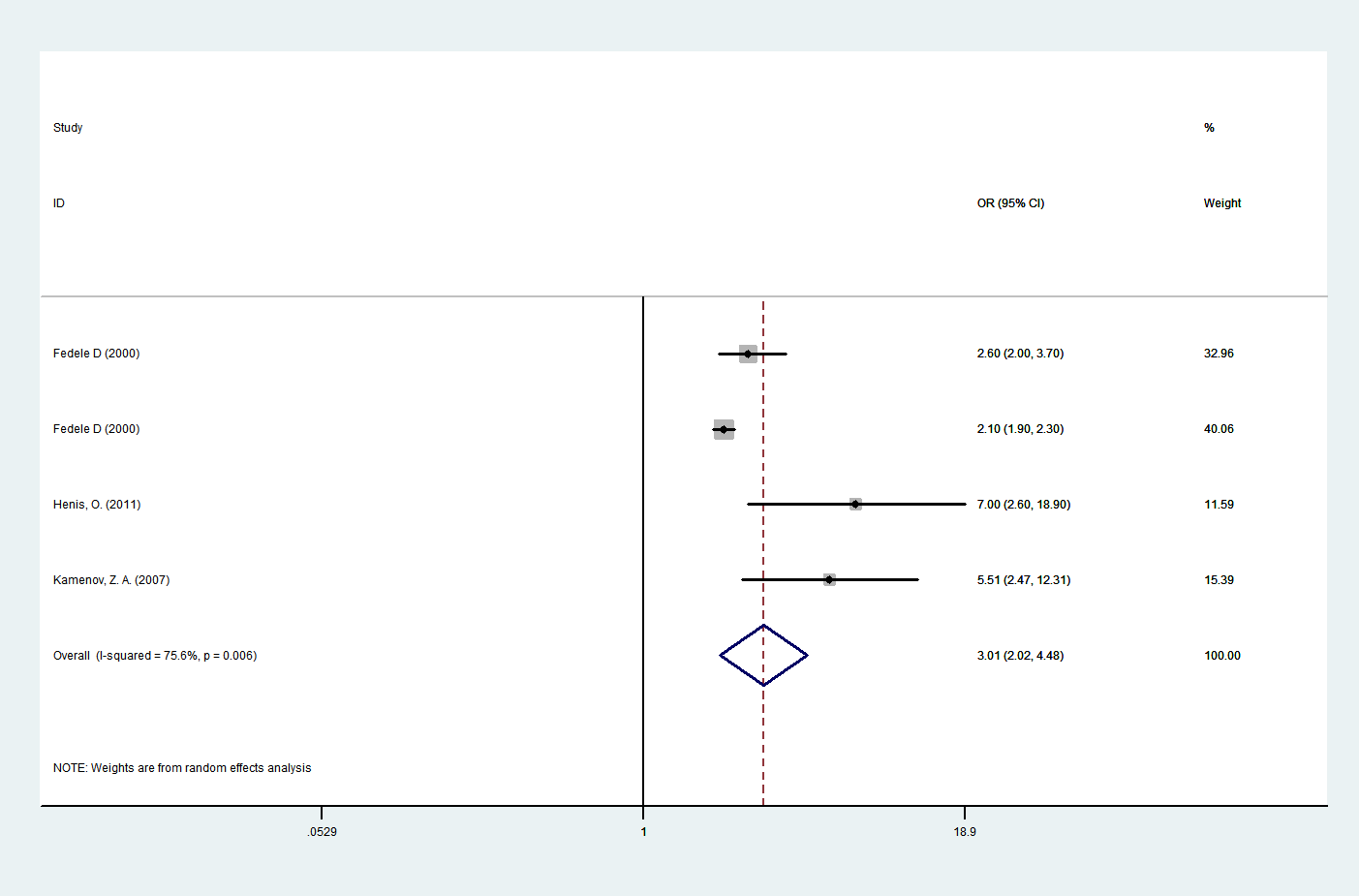


**Supplementary Figure 25.**  Forest plot of the association between Diabetic retinopathy and Erectile Dysfunction in Diabetes Mellitus.


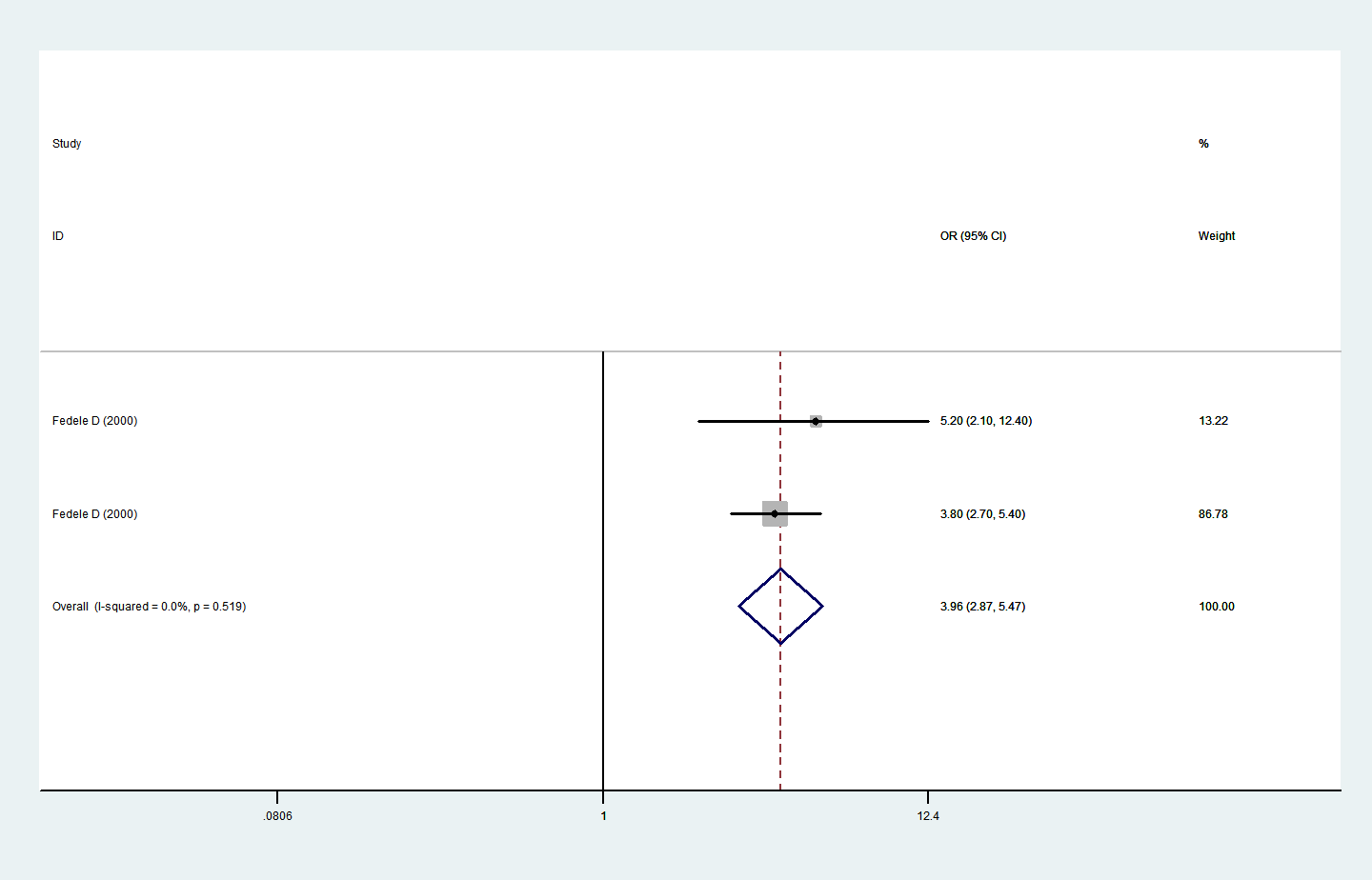


**Supplementary Figure 26.**  Forest plot of the association between Diabetic foot and Erectile Dysfunction in Diabetes Mellitus.


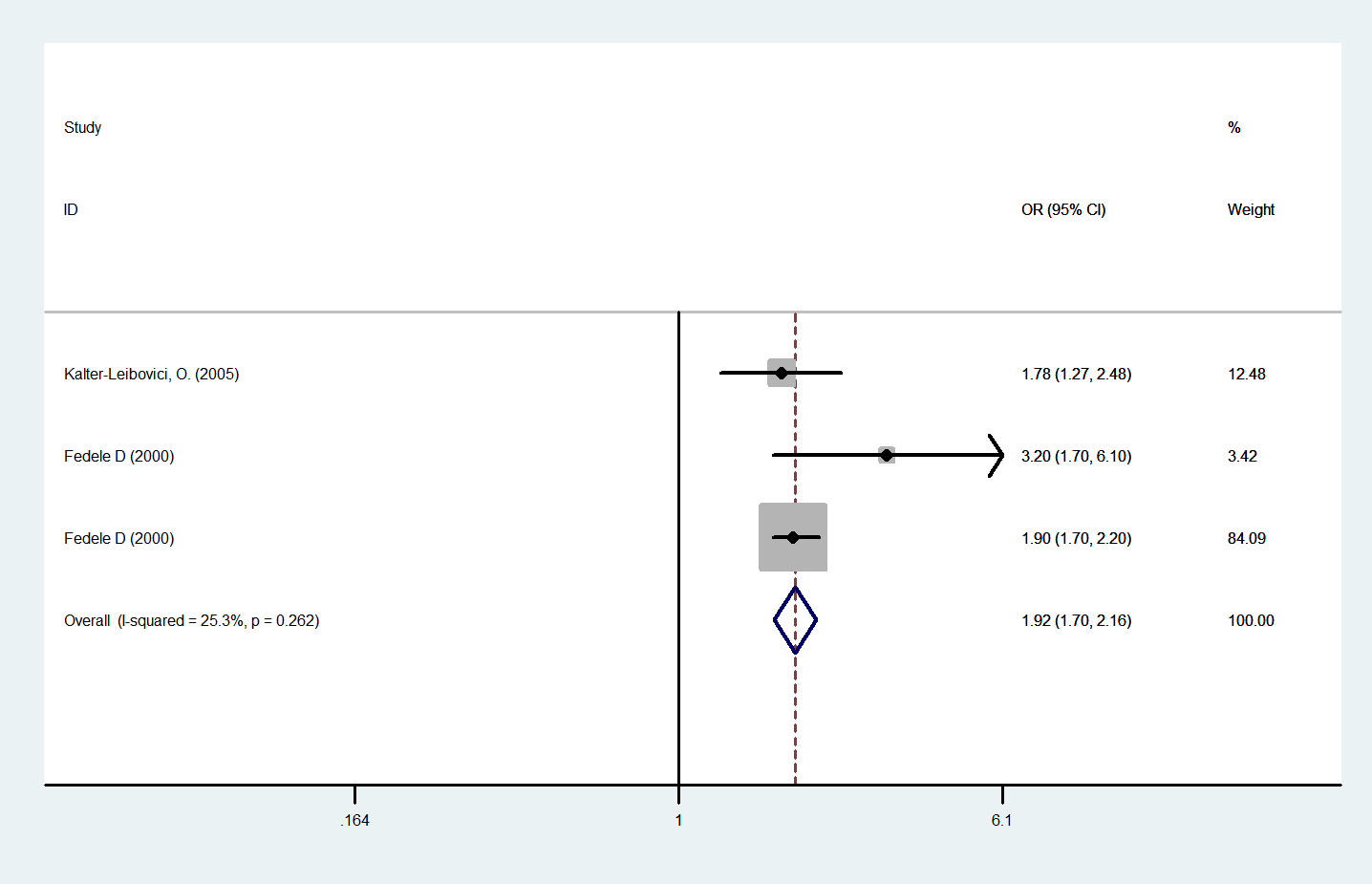


**Supplementary Figure 27.**  Forest plot of the association between Cardiovascular disease and Erectile Dysfunction in Diabetes Mellitus.


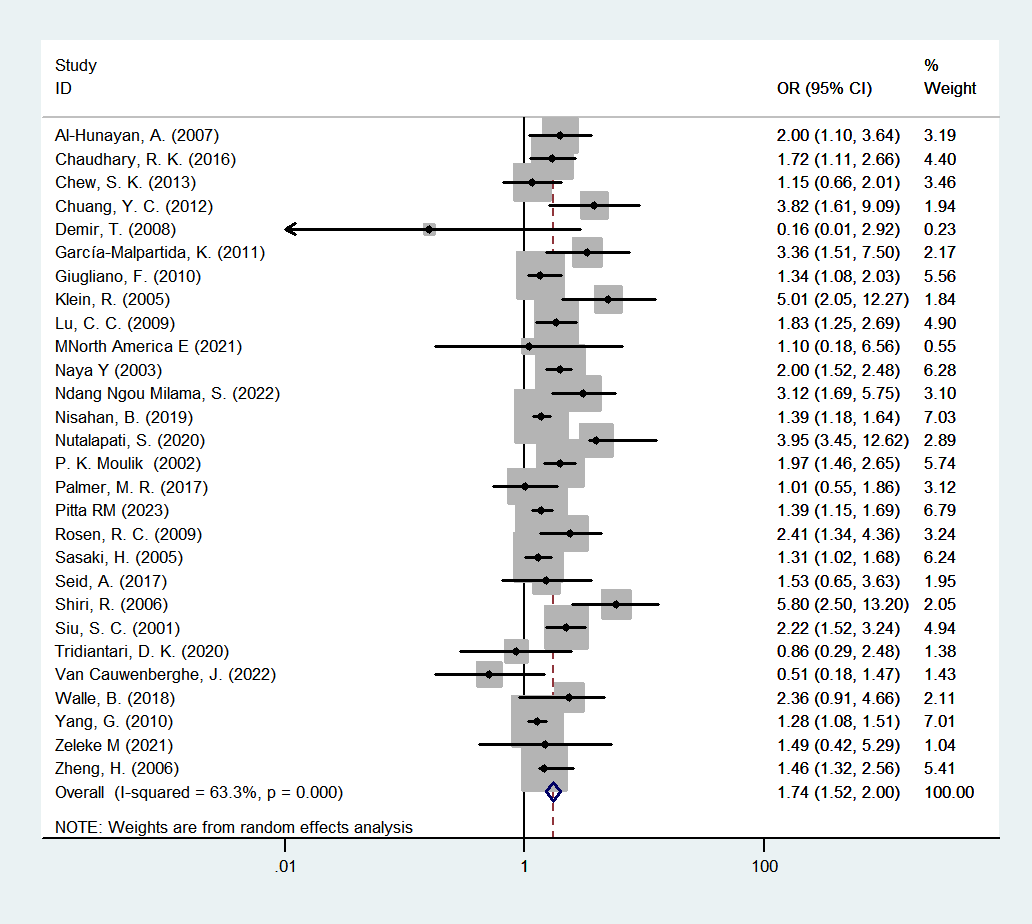


**Supplementary Figure 28.**  Forest plot of the association between Hypertension and Erectile Dysfunction in Diabetes Mellitus.


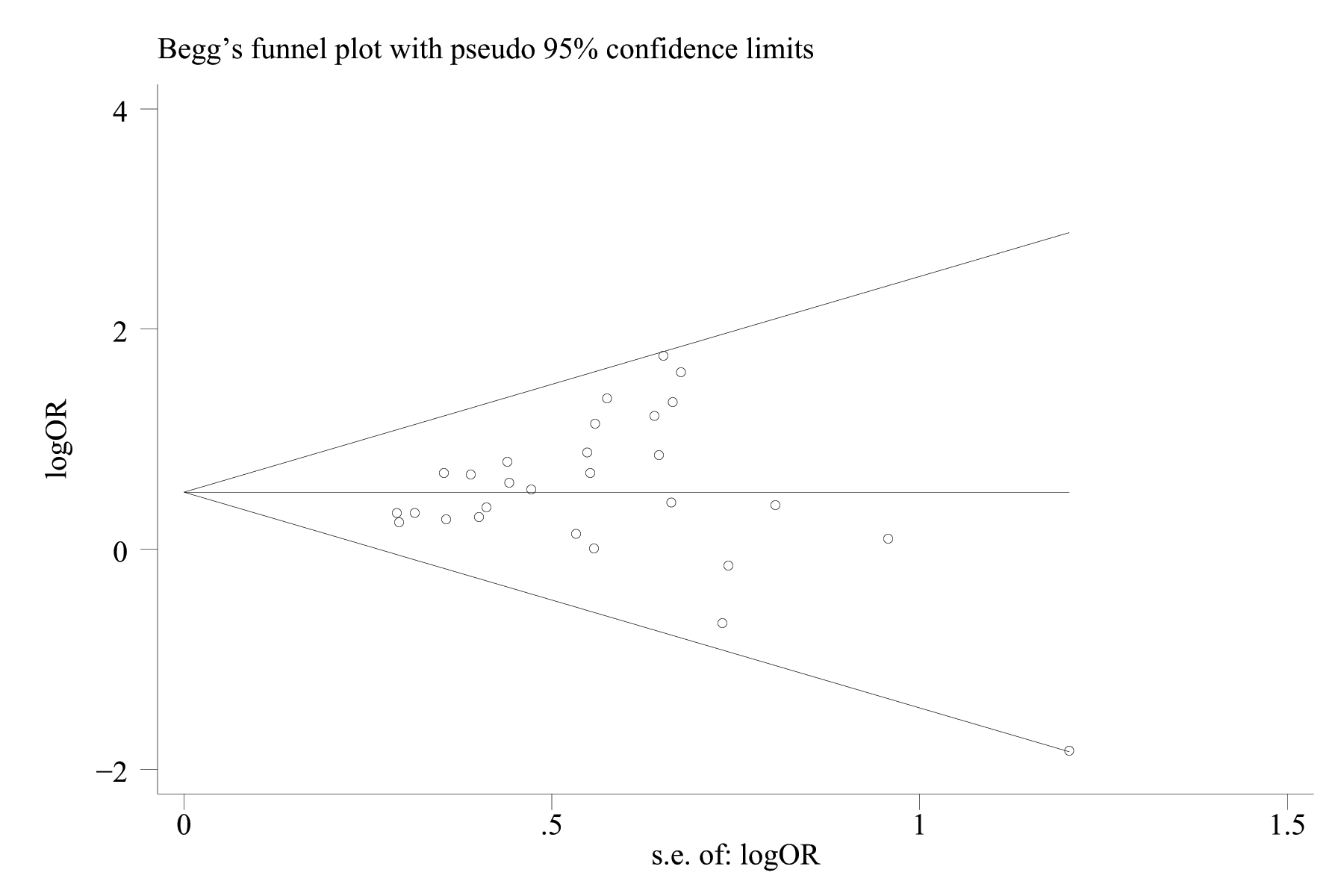


**Supplementary Figure 29.** Begg’s funnel plot for publication bias of Hypertension Duration of DM and Erectile Dysfunction in Diabetes Mellitus.


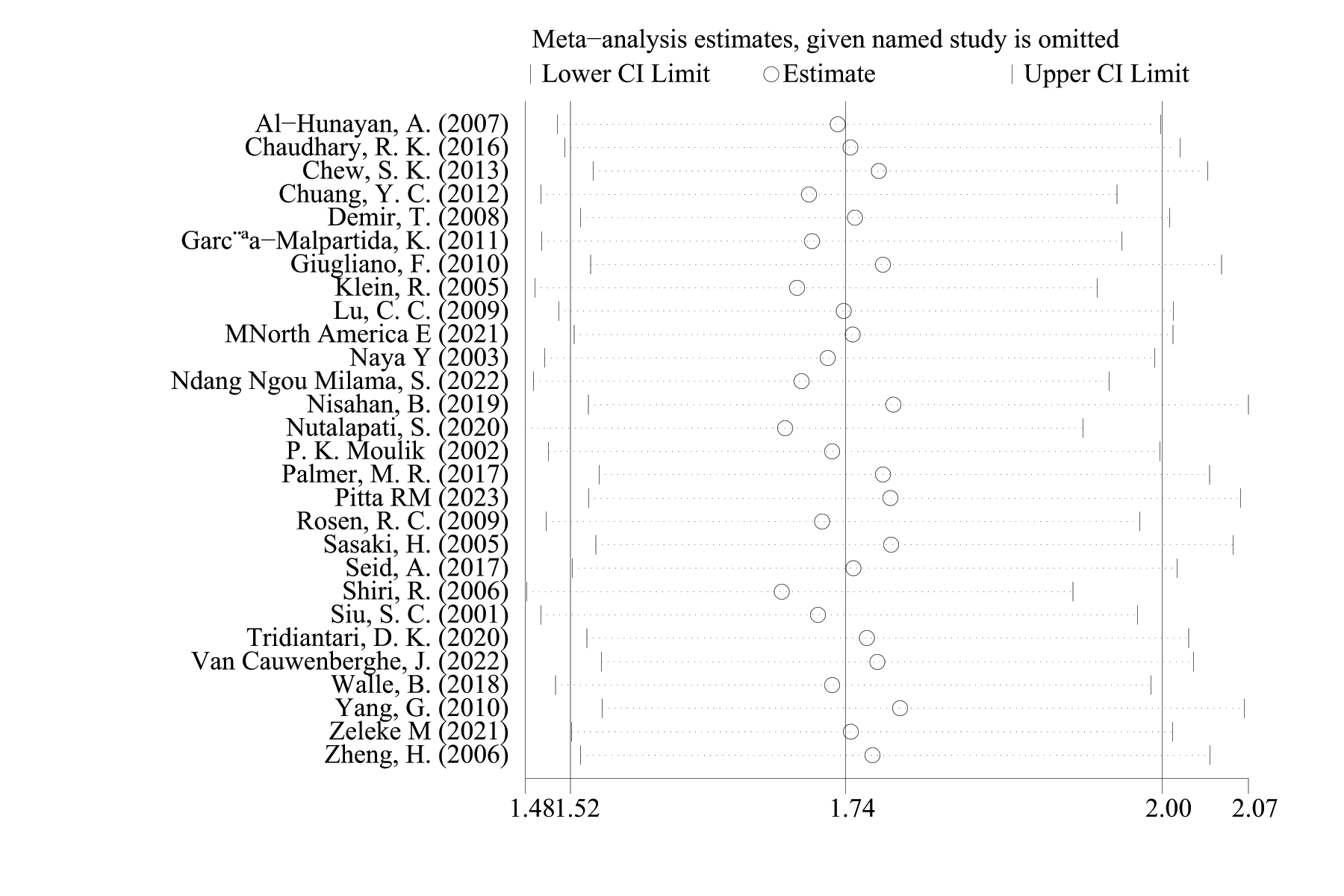


**Supplementary Figure 30.** Sensitivity analysis of the enrolled studies for associations between Hypertension and Erectile Dysfunction in Diabetes Mellitus.


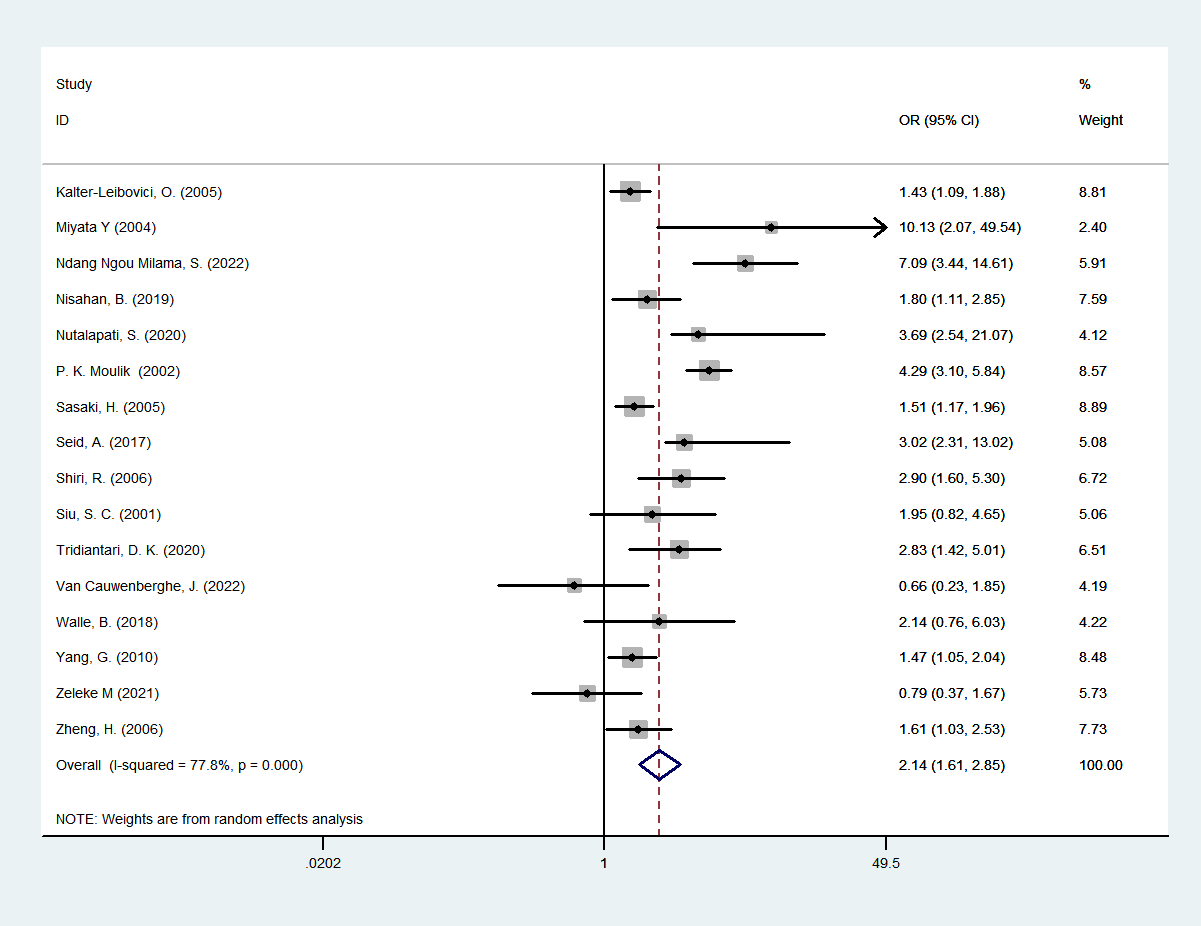


**Supplementary Figure 31.**  Forest plot of the association between Microvascular disease and Erectile Dysfunction in Diabetes Mellitus.


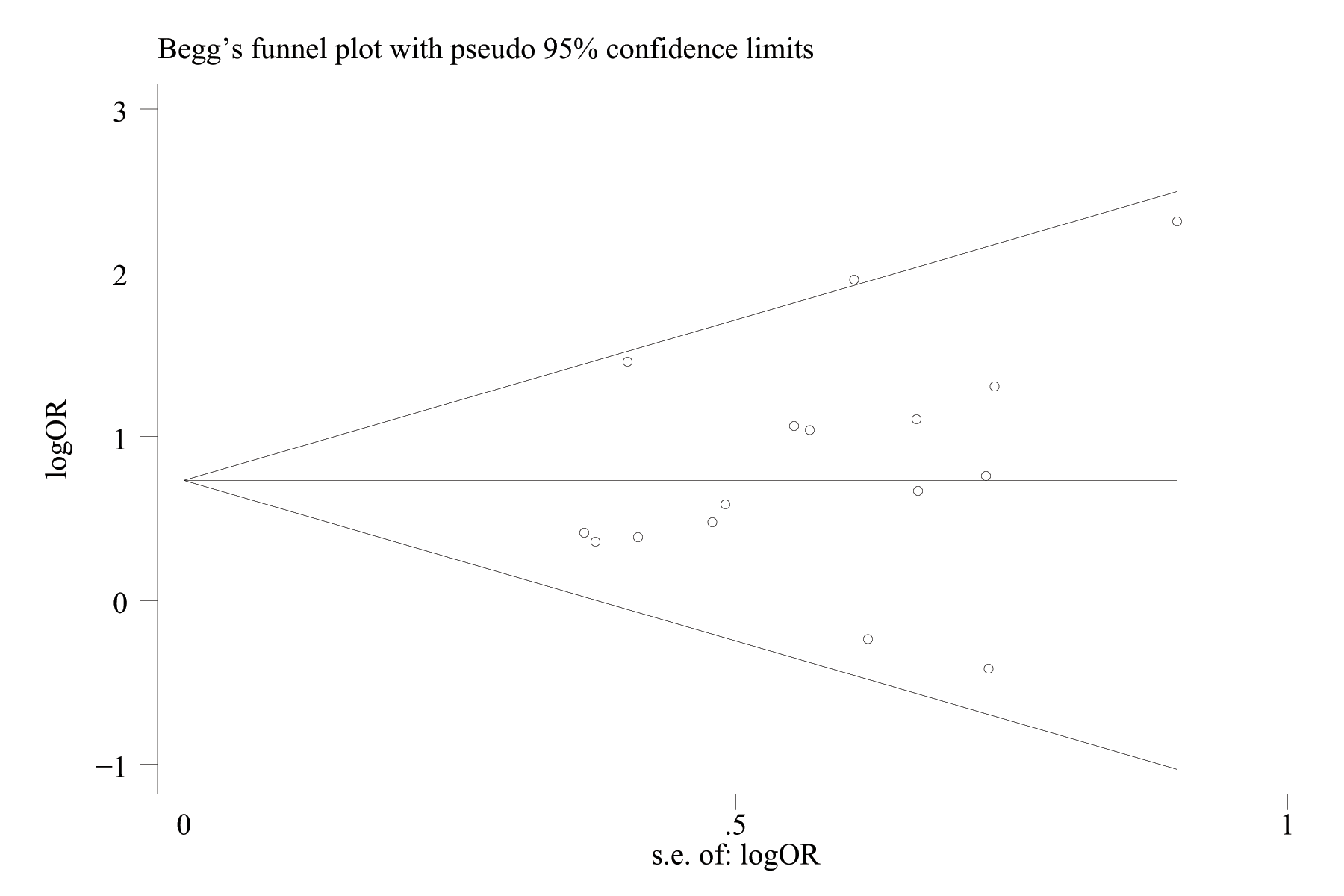


**Supplementary Figure 32.** Begg’s funnel plot for publication bias of Microvascular disease of DM and Erectile Dysfunction in Diabetes Mellitus.


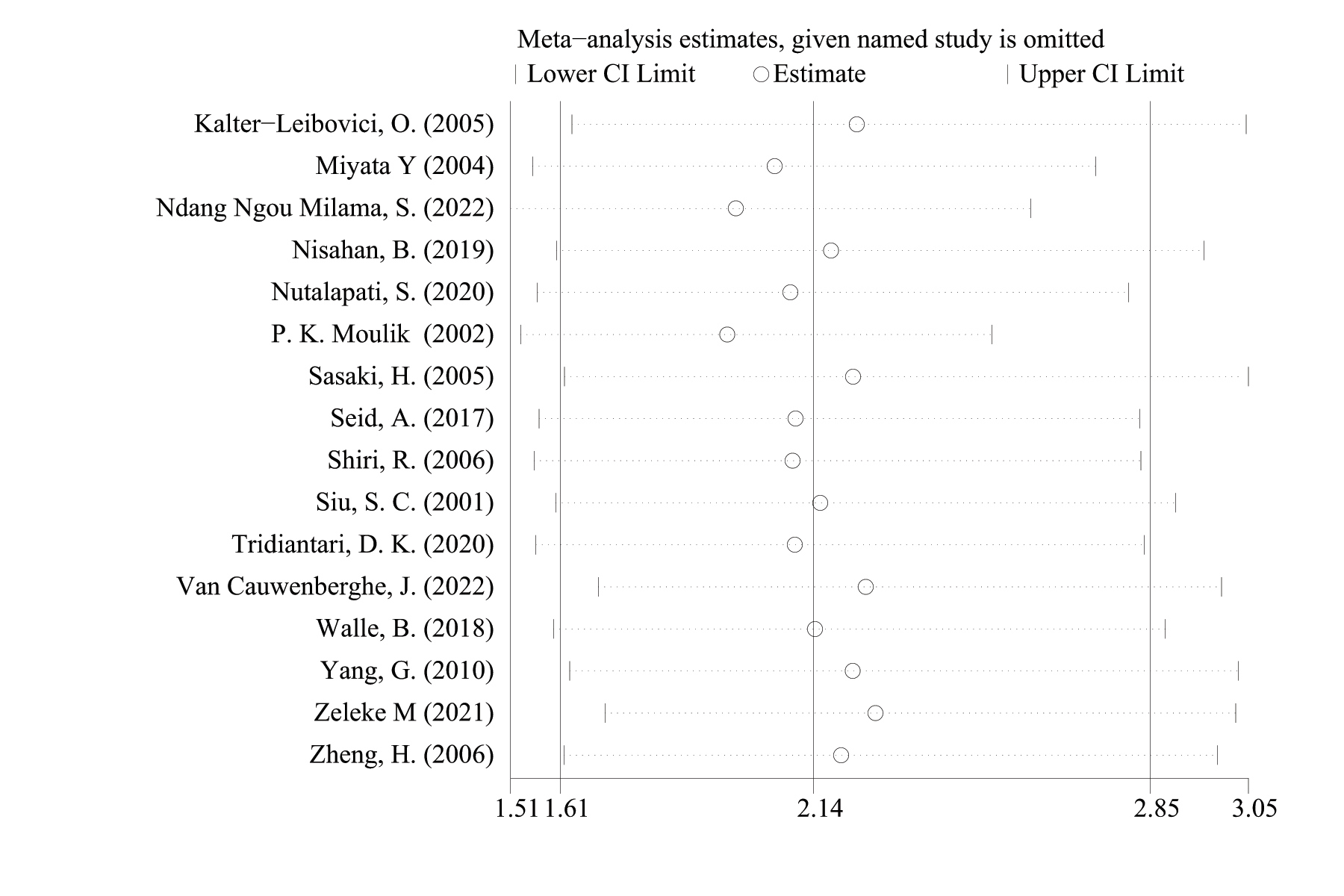


**Supplementary Figure 33.** Sensitivity analysis of the enrolled studies for associations between Microvascular disease and Erectile Dysfunction in Diabetes Mellitus.


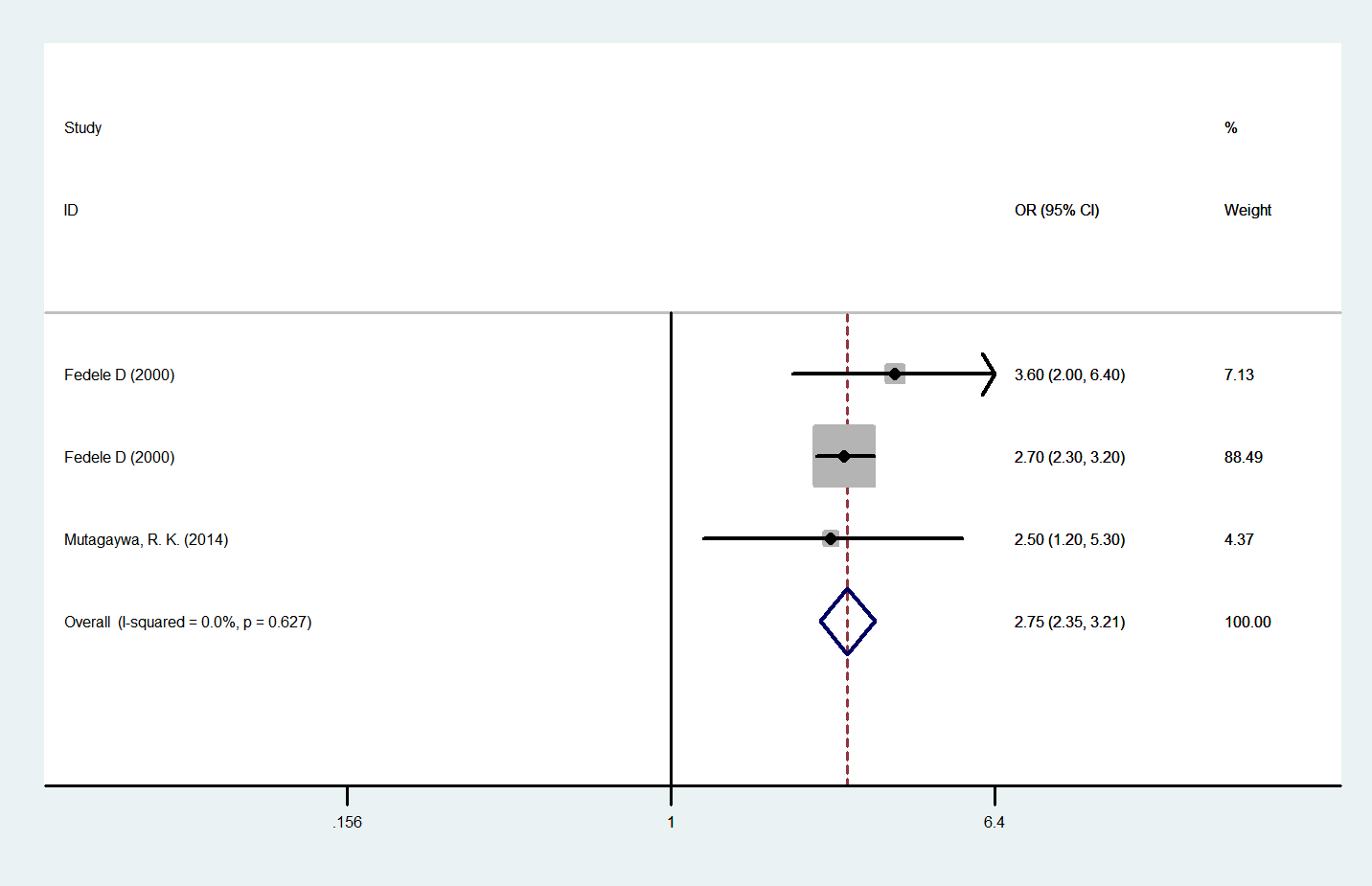


**Supplementary Figure 34.**  Forest plot of the association between Vascular disease and Erectile Dysfunction in Diabetes Mellitus.


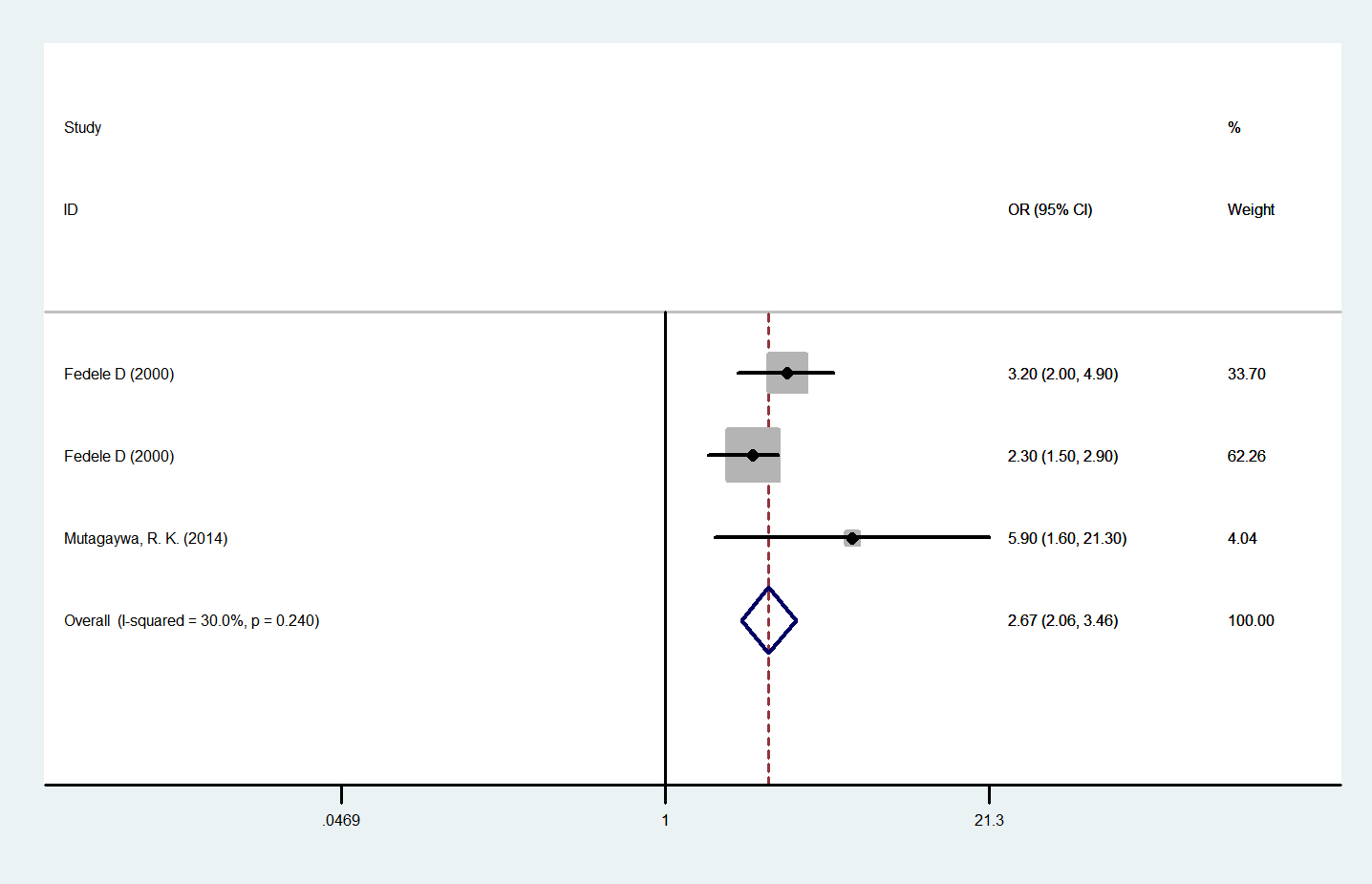


**Supplementary Figure 35.**  Forest plot of the association between Nephropathy and Erectile Dysfunction in Diabetes Mellitus.


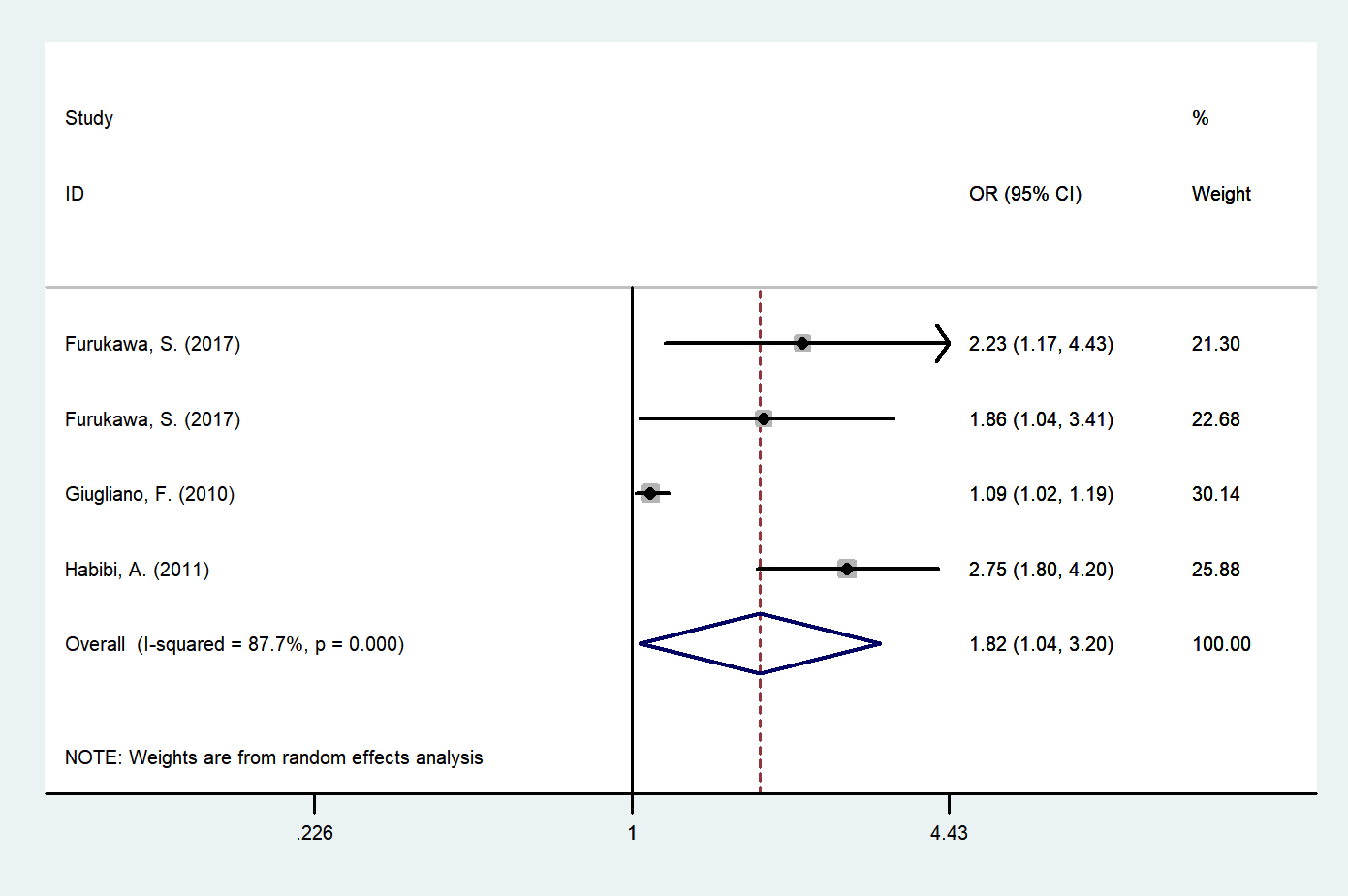


**Supplementary Figure 36.**  Forest plot of the association between Depression and Erectile Dysfunction in Diabetes Mellitus.


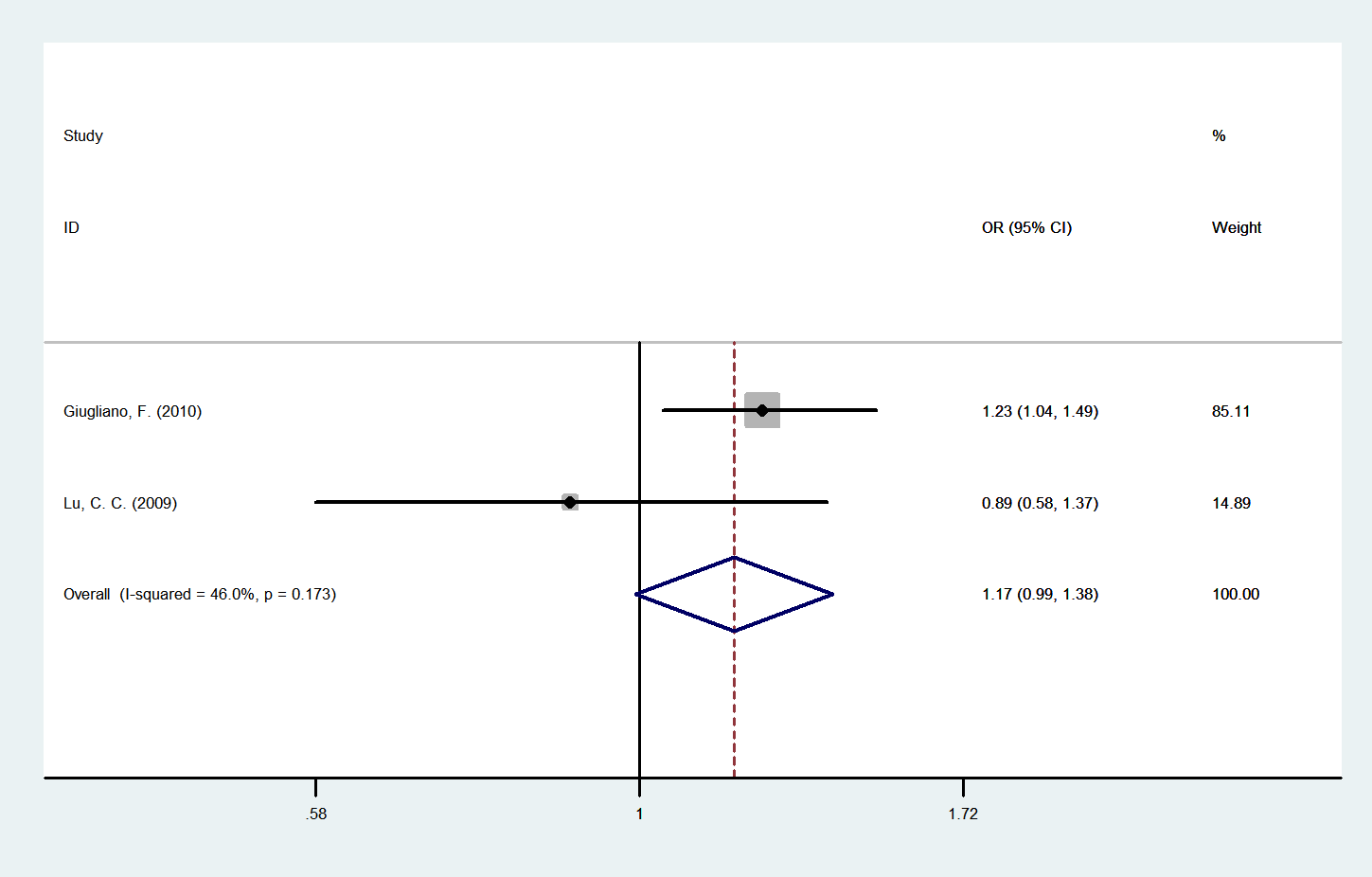


**Supplementary Figure 37.**  Forest plot of the association between Atherogenic dyslipidemia and Erectile Dysfunction in Diabetes Mellitus.


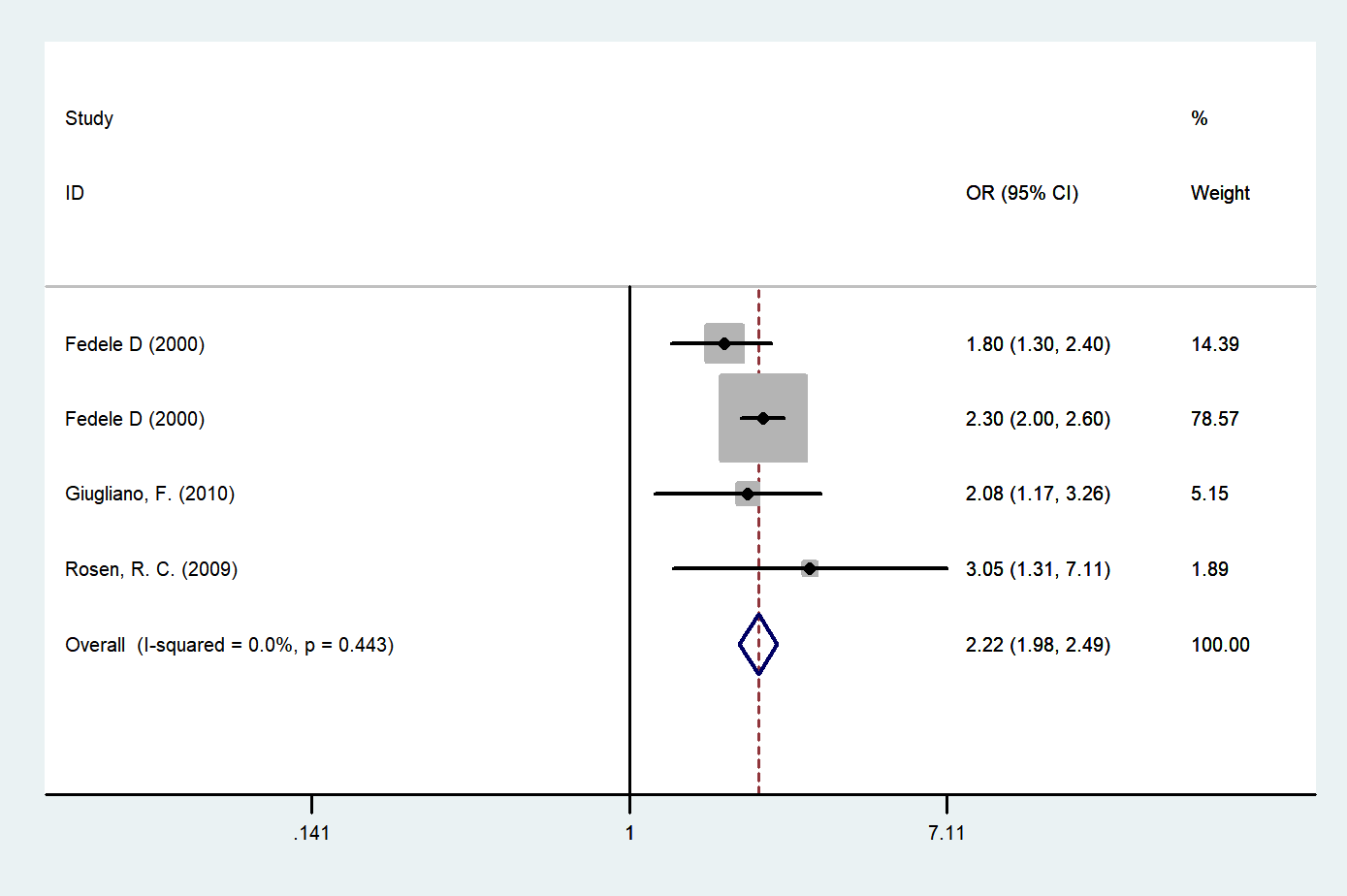


**Supplementary Figure 38.**  Forest plot of the association between Metabolic syndrome and Erectile Dysfunction in Diabetes Mellitus.
